# Cloning and Characterization of 1,8-Cineole Synthase (*SgCINS*) Gene From the Leaves of *Salvia guaranitica* Plant

**DOI:** 10.3389/fpls.2022.869432

**Published:** 2022-04-15

**Authors:** Mohammed Ali, Dikhnah Alshehri, Abeer Mousa Alkhaibari, Naeema A. Elhalem, Doaa Bahaa Eldin Darwish

**Affiliations:** ^1^Egyptian Deserts Gene Bank, North Sinai Research Station, Department of Genetic Resources, Desert Research Center, Cairo, Egypt; ^2^Department of Biology, Faculty of Science, University of Tabuk, Tabuk, Saudi Arabia; ^3^Department of Botany, Faculty of Science, Mansoura University, Mansoura, Egypt

**Keywords:** *Salvia guaranitica*, terpene synthase (TPS), transgenic *Nicotiana tabacum*, *Spodoptera littoralis* (cotton leafworm), 1-8-cineole synthase (*SgCINS*)

## Abstract

Monoterpenes are one of the most common groups belonging to the terpenoid family, with a C10 structure comprising of two isoprene units. Most of monoterpenes are volatile plant compounds, and they act as signaling molecules between plants and the environment, particularly as defensive compounds against herbivores and pathogens. In this study, 1,8-cineole synthase (*SgCINS*) gene was identified and cloned from the leaves of *Salvia guaranitica* plant. To examine the role of *SgCINS* in insect resistance, we transformed and expressed this gene into tobacco leaves. The metabolic analysis revealed that the production of various types and amount of terpenoid was increased and decreased in *SgCINS* overexpression and control lines, respectively, suggesting that overexpressing *SgCINS* in transgenic tobacco plants lead to an increase in the production of various types of terpenoids and other phytochemical compounds. These results indicated why transgenic tobacco was highly resistant against cotton worm than the highly susceptible control plants. Our results demonstrate that the *SgCINS* gene can play an important role in plants against cotton worm insect attack, and pave the way for using terpenoids genes for improving resistance to insect attack in higher plants.

## Introduction

Terpenes (i.e., terpenoids or isoprenoids) are considered the largest ecophysiologically active secondary metabolites with over 40,000 compounds known (Liu et al., 2014; Srividya et al., 2015; Ali et al., 2017, 2018, 2021). Terpenoids compounds have various structures and sizes and play numerous functional roles in primary and secondary metabolism (Rashad et al., 2022; Ali et al., 2021; Ali et al., 2017, 2018). In nature, terpenes play crucial roles in plant defense against a variety of organisms (Bakkali et al., 2008; Pavela and Benelli, 2016; Nobsathian et al., 2019; Rashad et al., 2022). In particular, their demonstrated toxicity against different insect pests makes terpenes suitable candidates for the development of eco-friendly pesticides (Isman, 2000; Miresmailli and Isman, 2014). Thus, various plant essential oils (EOs) and their terpenes have proven to have contact and fumigant toxic effects against mosquitoes (*Culiseta longiareolata*) (Maheswaran and Ignacimuthu, 2012; Pavela, 2015; Govindarajan et al., 2016), cotton leafworms (*Spodoptera littoralis*) (Liu et al., 2014), house flies (*Musca domestica*) (Rossi et al., 2012; Rossi and Palacios, 2013; Rossi and Palacios, 2015; Zhang et al., 2017; Benelli et al., 2017, 2018a,2018b), lepidoptera larvae (*Lepidoptera genitalia*) (Pavela, 2014), stored grain pests (e.g., *Tribolium castaneum, Rhyzopertha dominica, Sitophilus oryzae*, and *Sitophilus zeamais*) (Rajendran and Sriranjini, 2008; Herrera et al., 2015), and several other insect pests. The biological activity of plant EOs depends on the qualitative and quantitative analyses of terpene components (Bekele and Hassanali, 2001; Pavela, 2008).

*Salvia* is a genus from the Lamiaceae family, in which 1,000 species are distributed in Central and South America, East Asia, and West Asia, while the other species are distributed throughout the world (Alziar, 1988/1993; Ali et al., 2017, 2018; Sarrou et al., 2017). Many of salvia species are used in Chinese folk medicine, some of which are considered endemic, cultivated, or wild, including, *Salvia przewalskii, S. japonica, S. fruticosa, S. tuxtlensis, S. epidermindis, S. miltiorrhiza, S. aureus, S. santolinifolia, S. hydrangea, S. nipponica, S. tomentosa, S. lavandulifolia, S. chloroleuca, S. glabrescens, S. isensis, S. allagospadonopsis, S. macrochlamys*, and *S. recognita*. Salvia EO is composed of a mixture of terpenoids (i.e., mono-, sesqui-, di-, ses-, and triterpenoids), which are released from different parts of these species and play crucial roles in plant especially in plants’ defense against insect herbivores (Liu et al., 2014; Ali et al., 2017, 2018; Sarrou et al., 2017).

Plant genetic transformation with desired genes encoding factors that are related to resistance to phytophagous insects is a novel and attractive alternative from the synthetic chemical insecticides in the control of various aggressive plant pests (Meeusen and Warren, 1989; Estruch et al., 1997). Over the years, *Agrobacterium tumefaciens*-mediated transformation system has become a powerful tool to investigate the function of genes that are involved in primary and secondary metabolism (Wu et al., 2008; Zhang et al., 2013; Ali et al., 2017, 2018). Moreover, various studies have successfully used this system to uncover the role of different genes, such as *GmAOS* and *GmNES*, encoding allene oxide synthase and a novel monoterpene synthase, respectively, from soybean and determined the roles of these genes to protect plants against insect attack (Wu et al., 2008; Zhang et al., 2013). In this study, we performed the characterization and cloning of *SgCINS* gene involving 1,8-cineole synthase from *S. guaranitica* and determined its biological role in resistance against cotton worm through force-feeding-preference tests. These results suggest that overexpression of *SgCINS* gene in transgenic tobacco may be a better way of enhancing tolerance against the cotton worm insect attack.

## Materials and Methods

### Plant Materials and Tissue Collection

Seedlings of *S. guaranitica* L. were collected from the Wuhan Botanical Garden, China, and grown at National Research Center, Cairo, Egypt. Different tissues were sampled from 2-year-old *S. guaranitica* plants. For gene cloning, four biological replicates from leaves were sampled and handled. Each replicate consisted of three young and three old leaves from the same plant. In addition, for quantitative real-time PCR (qRT-PCR), three biological replicates were collected from the following six parts (i.e., young leaves, old leaves, stems, bud flowers, flowers, and roots). All samples were directly frozen in liquid nitrogen and then stored at −20°C until RNA extraction.

### Bioinformatics Analysis of 1,8-Cineole Synthase (*SgCINS*) Gene From *S. guaranitica*

The nucleotide sequence of *SgCINS* was selected from our previous RNA-Seq data (Ali et al., 2018). The potential transit peptide and physiochemical properties were analyzed for *SgCINS* gene using the PROTPARAM software^1^ and bioinformatics tool^2^. Furthermore, a phylogenetic tree of *SgCINS* gene was constructed using PhyML---Phylogeny.fr website^3^. Moreover, the multiple sequence alignment was carried out using the Clustal Omega software with default parameters^4^. Putative tissue expression profile from forty-nine *Arabidopsis* tissues (e.g., dry seed, imbibed seed, 24 h, 1st node, flower stage 12, stamens, Cauline leaf, cotyledon, root, entire rosette after, transition to flowering, flower stage 9, flower stage 10/11, flower stage 12, flower stage 15, flower stage 12-carpels, flower stage 12-petals, flower stage 12-sepals, flower stage 15-carpels, flower stage 15-petals, flower stage 15-sepals, flower stage 15-stamen, flowers stage 15-pedicels, leaf 1 + 2, leaf 7, petiole, leaf 7, distal half, leaf 7, proximal half hypocotyl, root, rosette leaf 2, rosette leaf 4, rosette leaf 6, rosette leaf 8, rosette leaf 10, rosette leaf 12, senescing leaf, shoot apex-inflorescence, shoot apex-transition, shoot apex-vegetative, stem, 2nd internode, mature pollen, seeds stage 3 w/siliques, seeds stage 4 w/siliques, seeds stage 5 w/siliques, seeds stage 6 w/o siliques, seeds stage 7 w/o siliques, seeds stage 8 w/o siliques, seeds stage 9 w/o siliques, seeds stage 10 w/o siliques, vegetative rosette, stem epidermis top of stem, and stem epidermis bottom of stem) were extracted from the public RNA-Seq Atlas of Arabidopsis^5^. Putative subcellular localization of *SgCINS* gene products from *S. guaranitica* was inferred from their sequence similarity to characterized *Arabidopsis* protein at the Arabidopsis Information Resource^6^. Subcellular localization profile images were built using Cell Electronic Fluorescent Pictograph Browsers (Cell eFP^7^).

### RNA Extraction and cDNA Synthesis

Total RNA from four biological replicates of *S. guaranitica* were extracted from the leaf for gene cloning using the TRIzol reagent (Invitrogen, CA, United States) according to the manufacturer’s methods and instructions. Moreover, total RNAs from three biological replicates from each of the plant parts (e.g., young leaves, old leaves, stems, bud flowers, flowers, and roots) were extracted for qRT-PCR. Additionally, total RNAs from three biological replicates for each transgenic tobacco lines were extracted for semiquantitative RT-PCR. Total RNA samples were treated with DNase I (Takara, China). RNA quality was examined on 1.2% agarose gels, and the purity and concentration were analyzed using a Nano-Photometer spectrophotometer (IMPLEN, CA, United States). cDNA synthesis for gene cloning and qRT-PCR was performed with a 10 μg total RNA pool produced by mixing equal volumes of the three RNA replicates in a tube using a commercial reverse transcription kit (M-MLV, China) according to the manufacturer’s protocol (Hussain et al., 2017).

### QRT-PCR and Semiquantitative RT-PCR Analysis

Quantitative RT-PCR was performed by an IQ5 Multicolor Real-Time PCR Detection System (Bio-Rad, United States) as described previously (Ali et al., 2017, 2018, 2021; Hussain et al., 2017) with SYBR Green Master (ROX) (Newbio Industry, China) following the manufacturer’s instructions at a total reaction volume of 20 μl. A gene-specific primer for *SgACTIN* forward 5′-TGGTTGTGACTTTTGGTCC CA-3′ and reverse 5′-ACAAACCCACGCTTGAGATCC-3′ was used as a reference gene with 155 bp, and *SgCINS* forward 5′-CTC ACAGCTCTTGGATTCAG-3′ and reverse 5′-GGAAAGATGC TTCGTAGAGTT-3′ with 152 bp length; all the primers were designed using the primer designing tools of IDTdna^8^, while semiquantitative real-time PCR was performed on an Eppendorf PCR (Mastercycler Nexus PCR Machine from Eppendorf, United Kingdom) system with a total reaction volume of 25 μl. A gene-specific primer for *NtEF-1*α forward 5′-TGGTTGTGACT TTTGGTCCCA-3′ and reverse 5′-ACAAACCCACGCTTGAGA TCC-3′ was used as a reference gene with 155 bp and *SgCINS* forward and reverse. The semiquantitative RT-PCR conditions were as follows: predenaturation step at 95°C for 4 min, 35 cycles of amplification (95°C for 30 s, 60°C for 30 s, and 72°C for 1 min), and a final extension step at 72°C for 10 min. The PCR products for semiquantitative RT-PCR were resolved on 1.6% agarose gel, and the expression levels of *NtEF-1*α and *SgCINS* genes were detected.

### Cloning of Full-Length *SgCINS* Gene

Full-length cDNAs of *SgCINS* (GenBank: KX893964.1) was obtained by PCR amplification with short and long gene-specific primers designed based on our transcriptome sequencing data (Ali et al., 2018). Leaf cDNA was used as a template for the first PCR, which was performed with short primers, such as *SgCINS* forward 5′-ATGTGTACCATTAGCATGCATGTATC-3′ and reverse 5′-TTACATTTCTTAAAACCGTGCTGG-3′ with the KOD-Plus DNA polymerase (Toyobo, Japan) with the following cycling conditions: an initial step of 4 min at 95°C followed by 34 cycles of denaturation for 10 s at 98°C; 30 s at 60.4°C and an extension for 2 min at 68°C, and a final extension step for 11 min at 68°C. The first PCR products were used as templates for PCR cloning using long primers, such as *SgCINS* forward 5′-GGGGACAAGTTTGTACAAAAAAGCAGGCTTC ATGTGTACCATTAGCATG-3′ and reverse 5′-GGGGACCA CTTTGTACAAGAAAGCTGGGTTTACATTTCTTAAAACCG -3′ with the and KOD-Plus DNA polymerase. The amplified PCR products were purified using the QIAEX II Gel Extraction Kit, China and cloned into the Gateway entry vector pDONR221 using BP Clonase (Gateway BP Clonase II Enzyme mix, Invitrogen). The resulting pDONR221 construct harboring target gene was sequenced, and the LR Clonase (Gateway LR Clonase Enzyme mix, Invitrogen) was used for recombination into the destination vector pB2GW7 for *Nicotiana tabacum* transformation. Sanger sequencing confirmed that all final constructs contained *SgCINS* cDNAs. The construct was introduced into *A. tumefaciens* strain “GV3101” by direct electroporation.

### *Nicotiana tabacum* Leaf Transformation Procedure and Preparation of Agrobacterium Cultures for Infection

The pB2GW7-*SgCINS* vector was introduced into *A. tumefaciens* strain GV3101 using the direct electroporation method. The transformation procedure was performed using the leaf disk method as described previously (Horsch et al., 1984; Lloyd et al., 1986; Burow et al., 1990; Ali et al., 2017) with a few modifications. The transgenic tobacco plants were regenerated from transformed leaf disk following selection on 55 mg/L hygromycin. More than 16 individual transgenic tobacco lines were generated and examined with RT-PCR for positive transgenic lines. The positive plants with good and healthy roots were transferred to the greenhouse for acclimatization. Finally, the transgenic tobacco plants were analyzed for the insect resistance assay and terpenoid profiling.

### Leaf Feeding Assay

Experiments were performed on transgenic tobacco and control plants as described in De las Mercedes Dana et al. (2006), Singla-Pareek et al. (2006), Wu et al. (2008), and Liu et al. (2014) with a few modifications. For the force-feeding tests, four mature full green leaves from transgenic tobacco and control plants with nearly similar size were placed on moist filter paper in Petri dishes, and ten third-instar larvae of cotton leafworms (*Prodenia litura*, Fabricius) were placed on the leaf surface and allowed to feed on transgenic tobacco and control leaves. The relative growth rate (RGR) = (the weight of cotton worm after fed by leaf—the weight before fed)/(the weight of cotton worm before fed), which was monitored by three independent bioassays at different times (e.g., 0, 3, 6, 12, and 24 h) after the start of feeding.

### Metabolite Extraction From Transgenic and Non-transgenic Tobacco Leaves

All terpenoid compounds from transgenic tobacco leaves containing *SgCINS* gene and non-transgenic tobacco leaves (control) at different times (e.g., 0, 3, 6, 12, and 24 h) after the start of insect feeding were extracted and isolated. For this, leaves of transgenic tobacco and control were collected at different times after the start of feeding and were homogenized to a powder using liquid nitrogen with a pestle and mortar, and then the powder was soaked in amber storage bottles [60 ml screw-top vials with silicone/polytetrafluoroethylene (PTFE) septum lids]^9^, containing n-hexane as a solvent. Later, amber storage bottles were incubated at 37°C along with shaking at 210 rpm for 70 h. Then, the supernatant solvent was collected by centrifugation at 5,000 rpm and 4°C for 10 min, then pipetted into glass vials, and mixed with 1.5 ml of concentrated oils under a stream of nitrogen gas using an evaporator (Organomation; Toption-China-WD-12). The concentrated oils were transferred to a fresh 1.5-ml crimp vial amber glass and were placed on the autosampler of the gas chromatography mass spectrometry (GC-MS) system for the analysis as described previously by Ali et al. (2017, 2018, 2021).

### Statistical Analysis

Data for control and transgenic tobacco for the leaf feeding assay experiment were collected. The RGR was monitored by three independent bioassays at different times (e.g., 0, 3, 6, 12, and 24 h) after the start of feeding (Liu et al., 2014 and Wu et al., 2008). These data were analyzed using the SAS 9.2 software (SAS Institute, 2008).

## Results

### Bioinformatics Analysis of *SgCINS* Gene

**FIGURE 1:**
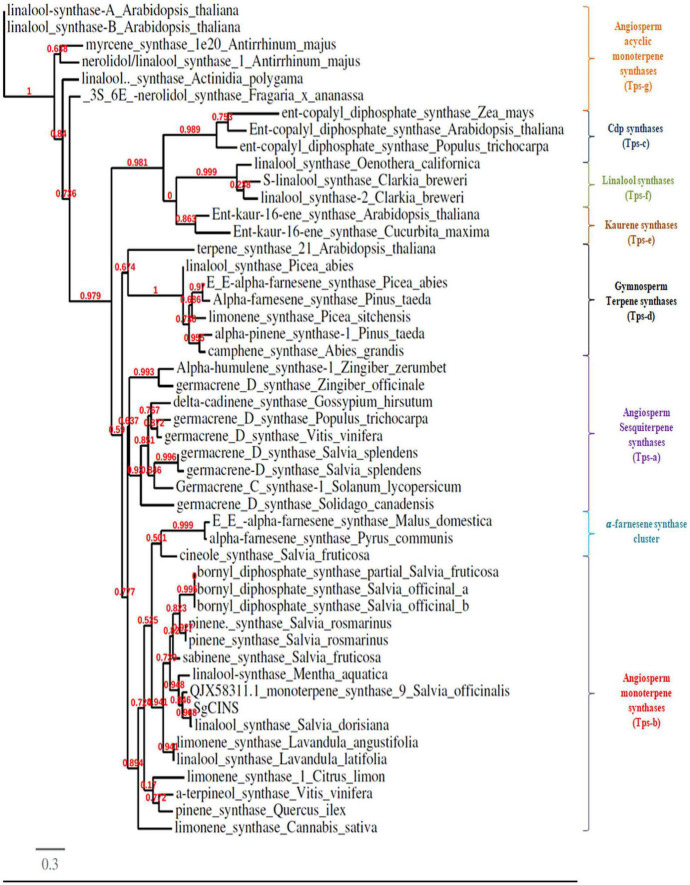
Phylogenetic tree of 1,8-cineole synthase (*SgCINS*) gene from *S. guaranitica* with selected terpene synthases genes from other plant species. Bootstrap values based on maximum likelihood are reported at the nodes.

**FIGURE 2:**
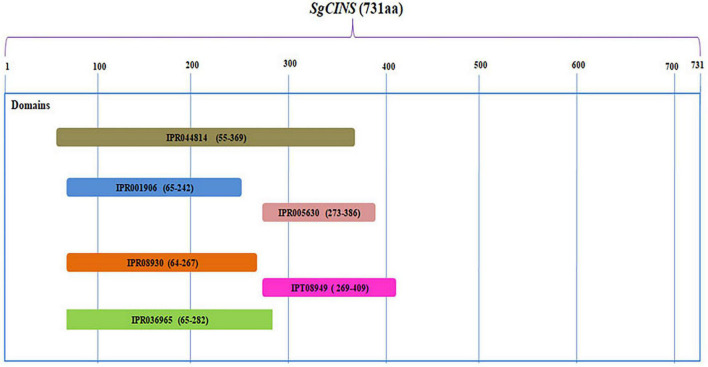
The putative domains structure for *SgCINS* gene. Six putative domains were detected in *SgCINS* at different amino acids positions such as, terpene cyclases, class 1, plant (IPR044814); terpene synthase, N-terminal domain (IPR001906); terpene synthase, metal-binding domain (IPR005630); terpenoid cyclases/protein prenyltransferase alpha-alpha toroid (IPR008930); isoprenoid synthase domain superfamily (IPT08949); and terpene synthase, N-terminal domain superfamily (IPR036965).

The complete ORF of *SgCINS* gene from *S. guaranitica* with 2,263 bp encoded a 731 amino acid protein with 86.636 kDa of molecular mass and 9.34 of predicted theoretical isoelectric point (pI). An analysis using the “iPSORT” program revealed that the *SgCINS* has a 30-amino-acid sequence (MCTISMHVSILSKPLNSLHRSERRSSNSWP) at the N-terminal, which means our target protein is localized in the chloroplast (i.e., plastid) and mitochondria, where terpene synthases (TPSs) are originated and their biosynthesis takes place. The phylogenetic tree was built using our target gene and TPS from Lamiaceae family and other plant species using PhyML-Phylogeny.fr website. The phylogenetic analysis result classified the *SgCINS* into the TPS-b subfamily of angiosperm monoterpene synthases (Figure 1). The putative function of *SgCINS* gene was initially predicted based on the sequence alignment with well-known other TPS sequences and conserved motifs from Lamiaceae family and other plants. Based on these predictions, *SgCINS* protein has various motifs such as RR (X8) W (residues 55–65), LYEAS (residues 194–199), RXR (residues 311–313), aspartate-rich-DDxxD (residues 348–352), and GTLxEL (residues 355–360) region, which are dominant at similar TPS genes (Zhao et al., 2021; Abbas et al., 2019; Su-Fang et al., 2014; Degenhardt et al., 2009; Ali et al., 2017, 2018; Supplementary Figure 1). Compared to other TPS genes, *SgCINS* has six domains that were specified by the InterPro protein sequence analysis and classification (InterPro^10^) database. So, *SgCINS* protein has six TPS family domains at different amino acid positions such as terpene cyclases, class 1, plant (IPR044814: from 55 to 369); TPS, N-terminal domain (IPR001906: from 65 to 242); TPS, metal-binding domain (IPR005630: from 273 to 386); terpenoid cyclases/protein prenyltransferase alpha-alpha toroid (IPR008930: from 64 to 267); isoprenoid synthase domain superfamily (IPT08949: from 269 to 409) and TPS, and N-terminal domain superfamily (IPR036965: from 65 to 282) (Figure 2). Finally, each protein sequence has one or two or all of these conserved domains belonging to the TPS family (Zhao et al., 2021; Abbas et al., 2019; Su-Fang et al., 2014; Degenhardt et al., 2009; Ali et al., 2017, 2018).

### Putative Tissue Expression Pattern and Subcellular Localizations of *SgCINS* Gene

The putative expression patterns of *SgCINS* gene of *S. guaranitica* were uncovered based on their higher similarity with *AT3G25830/TPS-CIN* gene from *A. thaliana*, and by transcript analysis across forty-nine *Arabidopsis* tissues (e.g., dry seed, imbibed seed, 24 h, 1st node, flower stage 12, stamens, Cauline leaf, cotyledon, root, entire rosette after, transition to flowering, flower stage 9, flower stage 10/11, flower stage 12, flower stage 15, flower stage 12-carpels, flower stage 12-petals, flower stage 12-sepals, flower stage 15-carpels, flower stage 15-petals, flower stage 15-sepals, flower stage 15-stamen, flowers stage 15-pedicels, leaf 1 + 2, leaf 7, petiole, leaf 7, distal Half, leaf 7, proximal half hypocotyl, root, rosette leaf 2, rosette leaf 4, rosette leaf 6, rosette leaf 8, rosette leaf 10, rosette leaf 12, senescing leaf, shoot apex-inflorescence, shoot apex-transition, shoot apex-vegetative, stem, 2nd internode, mature pollen, seeds stage 3 w/siliques, seeds stage 4 w/siliques, seeds stage 5 w/siliques, seeds stage 6 w/o siliques, seeds stage 7 w/o siliques, seeds stage 8 w/o siliques, seeds stage 9 w/o siliques, seeds stage 10 w/o siliques, vegetative rosette, stem epidermis top of stem, and stem epidermis bottom of stem) using the Arabidopsis Electronic Fluorescent Pictograph Browsers [eFP browsers (see text footnote 5)]. Interestingly, we observed the highest expression levels of this gene in seeds stage 10 w/o siliques, seeds stage 8 w/o siliques, seeds stage 9 w/o siliques, imbibed seed, 24 h, hypocotyl, stem epidermis bottom of stem, and stem epidermis top of stem (Supplementary Figures 2A,B). Moreover, we further explored the potential subcellular localization of the *SgCINS* gene products from *S. guaranitica* based on *Arabidopsis* protein localization to recognize possible synthesis sites using the Cell eFP browsers (see text footnote 7). From this analysis, the *SgCINS* gene is localized mainly in the plastid, followed by the mitochondrion and nucleus (Supplementary Figure 2C).

### Relative Expression Pattern of the *SgCINS* Gene by Quantitative RT-PCR

**FIGURE 3:**
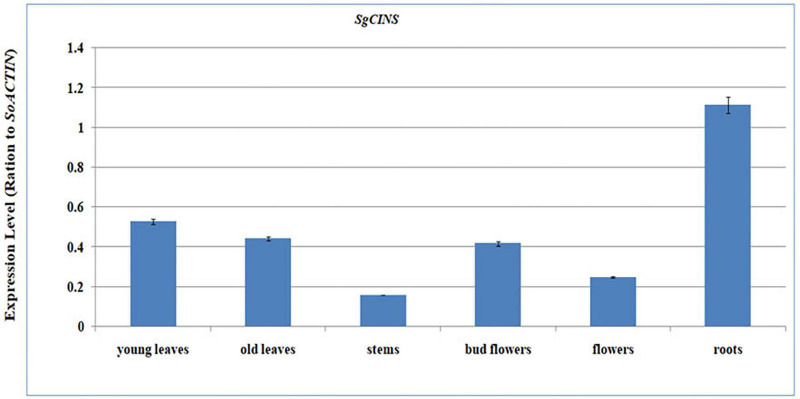
Quantitative RT-PCR validation of expression of *SgCINS* gene in *S. guaranitica*. Total RNAs were extracted from young leaves, old leaves, stem, bud flower, flower, and root samples, and the expression of *gCINS* gene was analyzed using quantitative RT-PCR. *SgACTIN* was used as the internal reference. The values are means ± SE of three biological replicates.

To determine the spatial expression patterns of the *SgCINS*, we quantified the expression levels of *SgCINS* transcripts in *S. guaranitica* young leaves, old leaves, stems, bud flowers, flowers, and roots tissues using the qPCR-PCR (Figure 3). From our results, we found that the *SgCINS* gene is expressed in all tissues with distinct expression patterns. In roots, *SgCINS* transcripts gene showed the highest expression levels, followed by young leaves, old leaves, bud flowers, flowers, and stems (Figure 3).

### Functional Characterization of *SgCINS* Gene in Transgenic *N. tabacum* Leaves and Insect-Resistance Analysis

**FIGURE 4:**
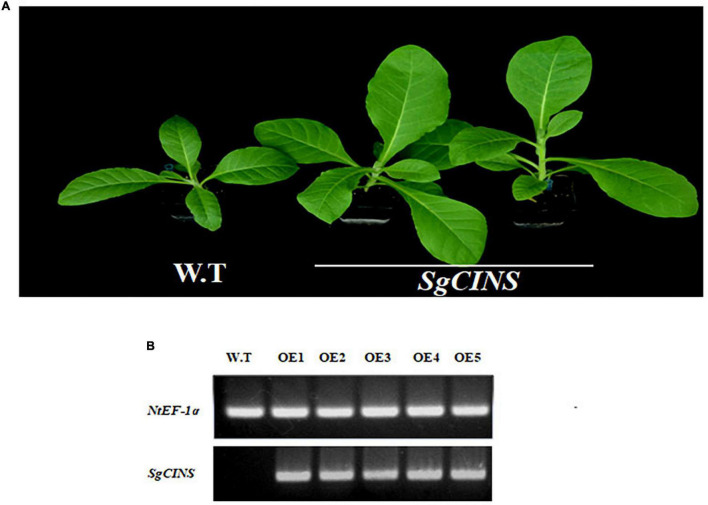
Overexpression of *S. guaranitica SgCINS* gene in transgenic tobacco. **(A)** Comparison of the phenotypes of the transgenic *N. tabacum* and wild-type *N. tabacum*. **(B)** Semiquantitative RT-PCR to confirm the expression of *SgCINS* gene.

**FIGURE 5:**
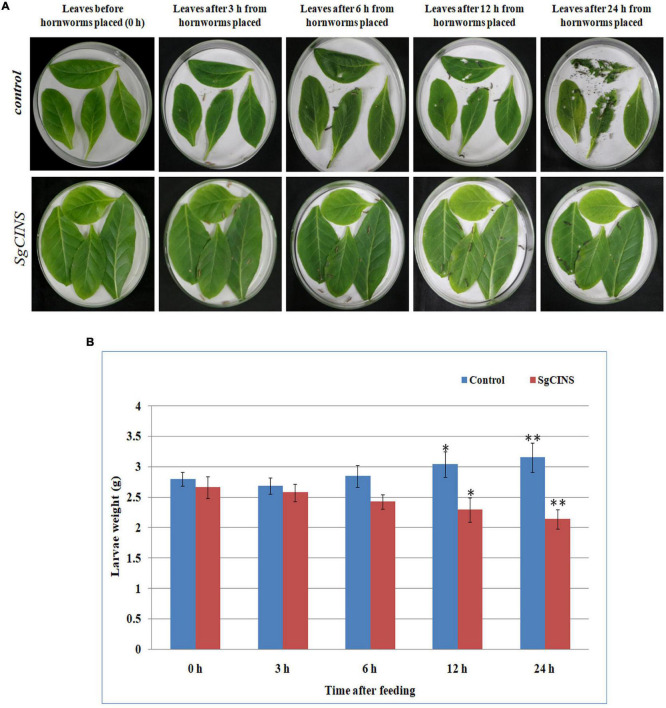
Force-feeding assays for insect resistance on control and transgenic plants after at different times (i.e., 0, 3, 6, 12, and 24 h). **(A)** Insect force-feeding assays with third-instar larvae of cotton leafworms (*Prodenia litura*) on *N. tabacum*. **(B)** The relative growth rate (RGR) for cotton leaf worms after feeding on control and T1 transgenic tobacco plants at different times (i.e., 0, 3, 6, 12, and 24 h). Each bar represents the mean and SD of the weight and significances are based on the Student’s *t*-test (*p < 0.05; ^**^p < 0.01).

To further investigate the function of *SgCINS* gene in plant insect resistance, *SgCINS* gene from *S. guaranitica* was cloned and overexpressed in tobacco as a transgenic expression system. The stable constitutive expression of the *SgCINS* gene in tobacco was carried out by the infection of *N. tabacum* leaves using *A. tumefaciens* strain GV3101 carrying pB2GW7-*SgCINS* under the control of 35S promoter (Figure 4A). Transgenic tobacco plants were successfully generated, which have many big green leaves with elliptical leaf shape, while the non-transgenic tobacco (i.e., control) showed a few numbers of green leaves with a lanceolate leaf shape (Figure 4A). The expression of the *SgCINS* gene in positive transgenic tobacco was confirmed using semiquantitative RT-PCR (Figure 4B). To investigate the role of *SgCINS* gene expression in transgenic tobacco in response to insect resistance, force-feeding-preference tests were conducted to evaluate the insect resistance of transgenic tobacco plants with *SgCINS* gene. Full green and healthy leaves with nearly similar sizes from transgenic and non-transgenic tobacco (i.e., control) plants were placed on a moist filter paper in Petri dishes, and ten third-instar larvae of cotton leafworms (*Prodenia litura*, Fabricius) were placed on the leaf surface and allowed to feed on transgenic tobacco or control leaves (Figure 5A). The RGR was documented after 0, 3, 6, 12, and 24 h of feeding. Most of cotton leafworms preferred to feeding on the non-transgenic tobacco leaves rather than the *SgCINS*-expressing tobacco leaves (Figure 5B), suggesting that some substances and compounds in transgenic tobacco had a negative effect on cotton leafworms.

### Detection of Terpenoids Compounds in Transgenic and Non-transgenic Tobacco Plants

**FIGURE 6:**
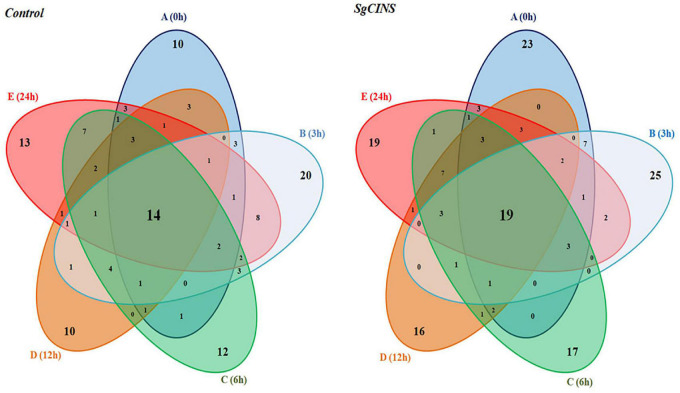
Five-way Venn diagram to show the number of unique and common compounds in the essential oil extracts from control and transgenic tobacco plants at different times from force-feeding assays for insect resistance [e.g., 0 h (A), 3 h (B), 6 h (C), 12 h (D), and 24 h (E)].

To gain insight into why transgenic tobacco plants were less damaged by the cotton worms, repelled the cotton leafworms, and inhibited their growth. So, we analyzed the terpenoids compounds from non-transgenic and transgenic tobacco plants overexpressing *SgCINS* at different times after the start of feeding using the *n*-hexane extraction method and GC-MS. The GC-MS analysis showed a significant difference between non-transgenic and transgenic tobacco plants in producing different amounts and types of terpenoids at different times after the start of feeding (Table 1, Figure 6, and Supplementary Table 1). As expected, the leaves of transgenic tobacco plants emitted a high level of various terpenoids compared with non-transgenic tobacco leaves. In this study, the improved production of different amounts and types of terpenoids at different times after the start of feeding was observed in transgenic tobacco plants that may be the reason for enhanced tolerance to cotton leafworms attack in transgenic tobacco. For GC-MS analysis, 283 compounds were identified using *n*-hexane extracts from non-transgenic and transgenic tobacco plants overexpressing *SgCINS* at different times (e.g., 0, 3, 6, 12, and 24 h) after the start of feeding. The numbers of compounds obtained from non-transgenic and transgenic tobacco leaves at different times (e.g., 0, 3, 6, 12, and 24 h) were 48 and 67 (96.36 and 100%), 67 and 58 (100 and 96.71%), 46 and 57 (92.13 and 100%), 47 and 64 (99.9 and 96.53%), and 65 and 62 (96.5 and 100%), respectively. The quantitative and qualitative analyses of all compounds from the *n*-hexane extraction are reported in Table 1 and Supplementary Table 1.

**TABLE 1:** The major chemical composition and terpenes from transgenic *Nicotiana tabacum* leaves at different times from insect force-feeding assays.

No.	Compound name	R.T	Formula	M.W/Da	Terpene of component	*W.T*					*SgCINS*				
						0 h	3 h	6 h	12 h	24 h	0 h	3 h	6 h	12 h	24 h
1	Artificial Almond Oil	9.524	C7H6O	106.1219	organic			0.38							
2	β-Cyclocitral	21.301	C10H16O	152.2334	mono										0.1
3	*trans*-p-Menth-1-en-3-ol	26.651	C10H18O	154.2493	mono										0.01
4	L-(-)-Nicotine	27.79	C10H14N2	162.232		17.98	14.22	12.69	8.59	7.97		0.18			
5	Heptacosane	27.949	C27H56	380.7335	alkane										0.01
6	Methyl perillate	30.187	C11H16O2	180.2435	mono						0.09		0.05	0.03	0.03
7	*trans*-β-Ionone	30.704	C13H20O	192.2973							0.08	0.07	0.05		0.07
8	Topanol;Stavox	31.447	C15H24O	220.3505	sesqui						3.33	0.08	1.15	0.7	0.61
9	(E)-β-Ionone	32.308	C13H20O	192.2973		0.07	0.07	0.1	0.05	0.06					
10	Actinidiolide, dihydro-	32.585	C11H16O2	180.2435	mono						0.03	0.05	0.02	0.01	0.03
11	Topanol;Stavox	33.083	C15H24O	220.3505	sesqui	0.4	0.12	0.62	0.21	0.22					
12	Agidol 3	33.514	C17H29NO	263.4183											0.01
13	(*trans*)-Carvyl propionate	35.114	C13H20O2	208.2967	mono									0.02	
14	1,4-Dimethyl-δ-3-tetrahydroacetophenone; 1,4-Dimethyl-3-cyclohexenyl methyl ketone;	35.78	C10H16O	152.2334	mono						0.04	0.07	0.04	0.03	0.03
15	Menthofuran; Menthofurane;p-Mentha-3,8-diene, 3,9-epoxy-	36.092	C10H14O	150.2176	mono									0.04	
16	Alpha.-Campholenal	36.134	C10H16O	152.2334	mono							0.04			
17	Germacra-1(10)	38.609	C15H20O2	232.3181	sesqui		0.05								
18	Palmitic acid, methyl ester	43.248	C17H34O2	270.4507	estre						1.12	0.14	0.09	0.08	0.14
19	Linolenic acid; α-Linolenic acid;	44.086	C18H30O2	278.4296		0.44	0.46	0.16	0.42	0.26					
20	Linolenic acid, methyl ester	44.131	C19H32O2	292.4562	estre						1.51	1.22	1.88	1.7	1.34
21	Linolenic acid; α-Linolenic acid;	44.155	C18H30O2	278.4296		0.2	0.22								
22	n-Hexadecanoic acid	44.777	C16H32O2	256.4241							6.5	7.47	6.13	6.06	6
23	Cetylic acid	44.922	C17H34O2	270.4507	estre		0.15	0.16	0.19						
24	γ-Linolenic acid	45.265	C18H30O2	278.4296		2.31	1.16		2.61	3.32					
25	Erucic acid;δ13-*cis*-Docosenoic acid; *cis*-13-Docosenoic acid;	45.374	C22H42O2	338.5677					1.08						
26	*trans*-Retinyl acetate	45.497	C22H32O2	328.4883		1.46	1.77	1.74	2.89	1.18					
27	Palmitic acid	45.96	C16H32O2	256.4241		15.28	6.84	5.36	13.1	13.86					
28	(+)-Ledol	46.00	C15H26O	222.3663	sesqui								0.6		
29	4,8,13-Duvatriene-1,3-Diol	46.045	C20H34O2	306.4828	diter						0.32	0.15		0.22	0.45
30	Retinol, acetate, all-*trans*-	46.156	C22H32O2	328.4883		1.1	0.85	0.72	1.33	1					
31	Retinyl acetate	46.365	C22H32O2	328.4883		1.61	1.94	1.76	2.86	1.46					
32	δ-Guajene	46.494	C15H24	204.3511	sesqui						0.36			0.29	0.47
33	geranylgeraniol	46.761	C22H36O2	332.52	diter							0.13			0.51
34	d-Ledol	46.804	C15H26O	222.3663	sesqui						0.4		0.58	0.3	
35	all-*trans*-Vitamin A acetate	47.018	C22H32O2	328.4883							0.37		0.7	0.31	0.57
36	Linoleic acid, methyl ester; Methyl *cis*,*cis*-9,12-octadecadienoate	47.264	C19H34O2	294.4721							4.51	0.13	0.4	0.22	0.66
37	Linolenic acid, methyl ester	47.404	C19H32O2	292.4562	estre							0.27	0.72	0.41	0.77
38	Linoleic; Linoleic acid; Linolic acid	47.42	C18H32O2	280.4455							5.29				
39	Phytol	47.70	C20H40O	296.531	diter						2.63	1.81	4.66	2.94	2.78
40	Globulol	47.795	C15H26O	222.3663	Sesgui			0.22	0.45	0.13					
41	4,8,13-Duvatriene-1,3-Diol	47.804	C20H34O2	306.4828	diter		0.17								
42	All-*trans*-Geranylgeraniol	47.998	C22H36O2	332.52	diter	0.27		0.4	0.51	0.17					
43	β-Elemol	48.015	C15H26O	222.3663	sesqui		0.34								
44	4,8,13-Duvatriene-1,3-Diol	48.126	C20H34O2	306.4828	diter								1.06	0.36	0.92
45	geranylgeraniol	48.149	C22H36O2	332.52	diter	3.65			7.19						
46	(-)-Globulol	48.169	C15H26O	222.3663	sesqui		4.99			3.35					
47	4,8,13-Duvatriene-1,3-Diol	48.181	C20H34O2	306.4828	diter						0.65	0.08			
48	geranylgeraniol	48.404	C22H36O2	332.52	diter	4.22	5.58	6.02	8.64	3.42			0.55	0.17	0.48
49	Widdrol	48.477	C15H26O	222.3663	sesqui	1.33			2.79						
50	(4aS,7S)-1,1,4a,7-Tetramethyl-2,3,4,4a,5,6,7,8-octahydro-1H-benzo[7]annulen-7-ol	48.482	C15H26O	222.366	sesqui					1.05					
51	steviol	48.495	C20H30O3	318.45	diter		1.72	1.82							
52	*cis*-Caryophyllene epoxide; *trans*-caryophyllene oxide	48.608	C15H24O	220.3505	sesqui	0.2									
53	4,8,13-Duvatriene-1,3-Diol	48.618	C20H34O2	306.4828	diter					0.2					
54	taraxastane	48.63	C30H50	410.718	triter		0.27	0.39	0.67						
55	Linolenic acid	48.938	C18H30O2	278.4296							13.31	14.71	11.27		10.2
56	Linolenic acid, methyl ester	49.069	C19H32O2	292.4562	estre				0.48						
57	Linolenic acid, ethyl ester	49.087	C20H34O2	306.4828	estre		0.66								
58	Linolenic acid, methyl ester	49.069	C19H32O2	292.4562	estre	0.48		0.36							
59	g-Elemene	49.284	C15H24	204.351	sesqui	3.82				2.98					
60	α-4,8,13-Duvatriene-1,3-Diol	49.3	C20H34O2	306.4828	diter		4.94	4.94	7.26						
61	Phytol	49.331	C20H40O	296.531	diter	5.2	3.86			3.65					
62	(-)-Ledol	49.631	C15H26O	222.3663	sesqui										0.68
63	Viridiflorine; (+)-Ledene	49.841	C15H24	204.3511	sesqui					1.44			0.66		
64	β-4,8,13-Duvatriene-1,3-Diol	49.836	C20H34O2	306.4828	diter	1.84	2.27	1.97	3.65						
65	α-Linolenic acid;	50.161	C18H30O2	278.4296		29.44	16.74	2.46	20.82	26.47					
66	Cedrol	50.227	C15H26O	222.3663	sesqui							0.07			
67	Labda-8(17),14-dien-6,13-diol	50.411	C20H34O2	306.4828	diter							1.49			
68	Ledol	50.518	C15H26O	222.3663	sesqui						13.02		22.49	14.42	20.61
69	α-Elemol; β-Elemol	51.059	C15H26O	222.3663	sesqui	0.3				0.24					
70	Epiglobulol	51.086	C15H26O	222.3663	sesqui		0.35	0.28	0.55						
71	*trans*-Lycopene	51.066	C40H56	536.8726	tetraterpene							0.33			
72	(+)-Ledol; d-Ledol	51.314	C15H26O	222.3663	sesqui				0.2						
73	Isoabienol	51.551	C22H36O2	332.52	diter								0.06		
74	4,8,13-Duvatriene-1,3-Diol	52.219	C20H34O2	306.4828	diter	0.92									
75	(+)-Ledol; d-Ledol	52.23	C15H26O	222.3663	Sesgui			3.09	5.35	0.97					
76	Sclareol	52.251	C20H34O2	306.4828	diter		2.78								
77	*cis*-Lycopene	52.395	C40H56	536.8726	tetraterpene							0.37			
78	Ascaridole	53.511	C10H16O2	168.2328	mono							0.11			
79	epi-α-Elemol	54.143	C15H26O	222.3663	sesqui								0.4		
80	Squalene	54.215	C30H50	410.718	triter						0.18			0.21	0.36
81	4,8,13-Duvatriene-1,3-Diol	54.733	C20H34O2	306.4828	diter							0.13			
82	4,8,13-Duvatriene-1,3-Diol	55.529	C20H34O2	306.4828	diter							0.12			
83	10-epi-Elemol	56.729	C15H26O	222.3663	sesqui			0.19							
84	Linolenic acid; α-Linolenic acid;	57.44	C18H30O2	278.4296									0.04		
85	Ethyl linolenate	61.85	C20H34O2	306.4828	estre								0.03		
86	Linolenic acid, methyl ester	61.956	C19H32O2	292.4562	estre						0.03				0.03
87	Ethyl α-linolenate	66.362	C20H34O2	306.4828	estre				0.03						
88	Isovaleric anhydride; iso-Pentanoic anhydride	68.826	C10H18O3	186.2481	mono										0.04
89	(-)-Myrtenol	73.15	C10H16O	152.2334	mono								0.04		
90	n-Hentriacontane; Untriacontane	73.916	C31H64	436.8399	alkane								0.02		
91	Tetrapentacontane	74.046	C54H110	759.4512								0.04			
92	Erucylamide;	74.807	C22H43NO	337.5829	amide								0.02		
93	n-Nonacosane	75.129	C29H60	408.7867	alkane	0.19									
94	Squalene	75.767	C30H50	410.718	triter						0.5	0.18	0.3	0.2	0.32
95	Linolenic acid; α-Linolenic acid;	78.087	C18H30O2	278.4296					0.03						
96	n-Nonacosane	79.364	C29H60	408.7867	alkane										0.29
	Total percentage (%) of monoterpenes										0.16	0.27	0.15	0.13	0.24
	Total percentage (%) of sesquiterpenes					6.05	5.85	4.4	9.55	10.38	17.11	0.15	25.88	15.71	22.37
	Total percentage (%) of diterpenes					16.1	21.32	15.15	27.25	7.44	3.6	3.91	6.33	3.69	5.14
	Total percentage (%) of triterpenes					0.27	0.39	0.67		0.68	0.18	0.3	0.41	0.68	0.27
	Total percentage (%) of tetraterpene											0.7			
	Total percentage (%) of alkane					0.19							0.09		0.01
	Total percentage (%) of ester					0.48	0.81	0.52	0.7		1.15	0.41	0.84	0.49	0.94

The identified compounds are listed based on the name of compounds, retention time, compounds mass, and percentage of peak area (Table 1 and Supplementary Table 1). First, in non-transgenic tobacco leaves, the sesquiterpene compounds were shown as the main group after 24 h (10.38%), followed by 12 h (9.55%), 0 h (6.05%), 3 h (5.85%), and 6 h (4.4%). Furthermore, the diterpene compounds were reported as the main group after 12 h (27.25%), followed by 3 h (21.32%), 0 h (16.1%), 6 h (15.15%), and 24 h (7.44%). In contrast, the triterpene compounds were shown as the main group after 24 h (0.68%), followed by 6 h (0.67%), 3 h (0.39%), and 0 h (0.27%). In addition, one alkane compound was detected after 0 h (0.19%). Moreover, the ester compounds were shown as the main group after 3 h (0.81%), followed by 6 h (0.52%), 3 h (0.48%), and 0 h (0.7%). Finally, the leaves of non-transgenic tobacco after all the times did not produce any monoterpene or tetraterpene compounds (Table 1 and Supplementary Table 1).

Second, in transgenic tobacco leaves overexpressing *SgCINS*, the monoterpene compounds were shown as the main group after 3 h (0.27%), followed by 24 h (0.24%), 0 h (0.16%), 6 h (0.15%), and 12 h (0.13%). Furthermore, after 6 h, the sesquiterpene compounds were observed to be the main group (25.88%), followed by 24 h (22.37%), 0 h (17.11%), 12 h (15.71%), and 3 h (0.15%). Moreover, the diterpene compounds were reported as the main group after 6 h (6.33%), followed by 24 h (5.14%), 3 h (3.91%), 12 h (3.69%), and 0 h (3.6%), while the triterpenes compounds were shown as the main group after 12 h (0.68%), followed by 6 h (0.41%), 3 h (0.3%), 24 h (0.27%), and 0 h (0.18%). Also, the tetraterpene compound group was detected only in transgenic tobacco leaves after 3 h (0.7%). After 12 h, the alkane compounds were reported as the main group (0.1%), followed by 6 h (0.09%) and 24 h (0.01%). Finally, the ester compounds were shown as the main group after 0 h (1.15%), followed by 24 h (0.94%), 6 h (0.84%), 12 h (0.49%), and 3 h (0.41%) (Table 1 and Supplementary Table 1). In contrast, the twelve hexane extracts from the non-transgenic and transgenic tobacco leaves at different times (e.g., 0, 3, 6, 12, and 24 h) after the start of feeding have unique, common, and major compounds (Figure 6). For example, the unique compounds in non-transgenic and transgenic tobacco leaves at different times were 10 and 23 (0 h), 20 and 25 (3 h), 12 and 17 (6 h), 10 and 16 (12 h), and 13 and 19 (24 h), respectively (Figure 6). Additionally, we found some other common compounds shared among all the times in non-transgenic and transgenic tobacco leaves (14 and 16), respectively.

## Discussion

### Characterization and Bioinformatics Analysis of *SgCINS* From *S. guaranitica* Plant

*Salvia* is considered one of the largest genera of plants in the Lamiaceae family. *Salvia* spp. terpenoids were analyzed several decades ago and were found to contain various types and amounts of mono-, sesqui-, di-, ses-, and triterpenes (Aziz et al., 2008; Loizzo et al., 2010; Fateme et al., 2013; Ali et al., 2017, 2018). Despite this, there are only a few number of recent reports that describe the role and the function of terpene genes in insect resistance (Zhang et al., 2013; Liu et al., 2014). In this study, we selected and identified the *SgCINS* gene from our RNA-Seq sequence data of *S. guaranitica* (Ali et al., 2018). The phylogenetic tree analysis showed that *SgCINS* from *S. guaranitica* forms a clade homology with linalool synthase from *S. dorisiana* and monoterpene synthase 9 from *S. officinalis*, and this clade belongs to angiosperm-specific TPS-b, which encodes to monoterpene synthase (Chen et al., 2011; Liu et al., 2014; Yu et al., 2020; Mehmood et al., 2021; Chen et al., 2021; Figure 1). To know the physiological role of *SgCINS*, we analyzed the putative expression patterns of our gene in forty-nine *Arabidopsis* tissues based on their higher similarity with *AT3G25830/TPS-CIN* gene from *A. thaliana*. This *SgCINS* gene was detected in all the tissues, and predominantly expressed in seeds stage 10 w/o siliques, seeds stage 8 w/o siliques, seeds stage 9 w/o siliques, imbibed seed, 24 h, hypocotyl, stem epidermis bottom of stem, and stem epidermis top of stem (Supplementary Figures 2A,B). *GmTPS21*, *SoHUMS*, *SoLINS2*, *SoNEOD*, *SgTPSV*, *SgFARD*, and *SgGERIS* genes from *G. max*, *S. officinalis*, and *S. guaranitica* were reported with higher expression levels in roots and seeds by Liu et al. (2014), Ali et al. (2017, 2018), respectively. Moreover, *SgCINS* was reported to be localized in the plastid (Supplementary Figure 2C). These results are in line with studies by Taniguchi et al. (2014), Chen et al. (2018), Ali et al. (2021), Wang et al. (2022) who reported that most of TPSs genes were targeted to the plastid or other cell organelles such as mitochondrion and nucleus. Additionally, an organ-specific expression pattern of *SgCINS* was determined using mRNA samples of 2-year-old *S. guaranitica* plants using the qRT-PCR. As shown in Figure 3, *SgCIN*S expressions were observed higher in roots (1.13-fold), young leaves (0.5-fold), old leaves (0.42-fold), and bud flowers (0.41-fold) rather than in the flowers (0.22-fold) and stems (0.18-fold). Similar results were obtained by Ali et al. (2017), of which the highest *SoCINS* expression was found in young leaves, followed by old leaves. We further cloned the full-length cDNAs of *SgCINS* from *S. guaranitica* and overexpressed this gene in transgenic tobacco leaves to evaluate the function of *SgCINS* gene in resistance to cotton leafworms.

### The Biological Functions of *SgCINS*

Terpenoids are considered from the major active secondary metabolites that may prevent insect and pathogen invasion by forming various chemical barriers (Dudareva et al., 2006; Chen et al., 2018). A number of terpenoid components from many plant species (e.g., *Commiphora erythraea; Valeriana jatamansi* Jones, *Valeriana officinalis* L., *Nardostachys chinensis* Bat, *Valeriana officinalis* L. var. *latifolia* Miq., *Oryza sativa*, and *G. max*) have been well studied, and most of them have been reported to be insect resistance such as, 1,8-cineole, nerol, (S)-limonene, geraniol, citronellal, citronellic acid, citronellol, linalool, (R)-limonene, (R)-α-pinene, (S)-β-pinene, (R)-pulegone, α-terpinene, γ-terpinene, thymol, camphene, and bornyl acetate (Zhang et al., 2013; Scalerandi et al., 2018; Chen et al., 2018; Feng et al., 2020; Muturi et al., 2020). *SgCINS*, identified as a 1,8-cineole synthase, was characterized to play an important role in the defense of transgenic tobacco plants against the third-instar larvae of cotton leafworms. In the leaf force-feeding tests, most cotton leafworms preferred the non-transgenic tobacco leaves over the *SgCINS*-expressing tobacco leaves. The significant differences in RGR of cotton worms (Figure 5B) indicate that the *SgCINS* overexpressors in transgenic tobacco had stronger resistance against cotton leafworms than non-transgenic tobacco, which had a weaker resistance against cotton leafworms, implying that they may be substances in these transgenic tobacco plants which interfered with the normal growth and development of cotton leafworms. It is sensible to attribute the various levels of resistance of transgenic and non-transgenic tobacco plants against cotton leafworms insect to the different types and amounts of terpenoids they produced (Figure 6 and Table 1).

### Overexpression of *SgCINS* Led to Changes the Accumulation of Different Type and Amounts of Terpenoids

A number of TPSs genes from a range of plant species have been well characterized, and most of them have the ability to convert the substrate into a single or diverse products during various reaction cycles, which are considered one of the unique traits of this type of gene (Aharoni et al., 2003; Yu and Utsumi, 2009; Ali et al., 2017, 2018; Chen et al., 2018). In this study, we found that the overexpression of *SgCINS* gene increased the accumulation of different types and amounts of terpenoids. Furthermore, the level of ester, not alkane, increased in overexpression tobacco plants. To understand the mechanisms underlying the *SgCINS*-related accumulation of terpenoids, we predicted the conserved motifs and domains using the InterPro protein sequence analysis and classification^11^ database (Ali et al., 2017, 2018). The amino acid sequence analysis indicated that *SgCINS* has various motifs [e.g., RR (X8) W, LYEAS, RXR, spartate-rich-DDxxD, and GTLxEL] and six domains at different amino acids positions (e.g., terpene cyclases, class 1, plant, TPS, N-terminal domain, TPS, metal-binding domain, terpenoid cyclases/protein prenyltransferase alpha-alpha toroid, isoprenoid synthase domain superfamily, and TPS, N-terminal domain superfamily). In previous studies, each protein sequence has one or two or some of these conserved motifs and domains belonging to the TPS family (Zhao et al., 2021; Chen et al., 2020; Abbas et al., 2019; Su-Fang et al., 2014; Degenhardt et al., 2009; Ali et al., 2017, 2018). As described above, this property is found in most of the characterized TPS synthases genes. For example, overexpression of *A. grandis* γ-humulene synthase, a sesquiterpene synthase gene with two DDxxD motifs that located on the opposite sides, can generate 52 compounds from sesquiterpenes (Steele et al., 1998; Ali et al., 2018). In addition, ectopic expression of rice *OsTPS19* can catalyze the formation of 16 compounds from monoterpenes such as, α-thujene, α-pinene, *cis*-ocimene, sabinene, myrcene, α-phellandrene, (S)-limonene, *trans*-ocimene, α-terpinene, γ-terpinene, *cis*-sabinene hydrate, *trans*-sabinene hydrate, α-terpinolene, terpinen-4-ol, neo alloocimene, and α-terpineol hydrate (Chen et al., 2018). In another example, (+)-sabinene synthase from *S. officinalis* catalyzes the formation of (+)-sabinene, terpinene, terpinolene, limonene, and myrcene in *in vitro* assays (Wise et al., 1998; Ali et al., 2018). Also, overexpression of lemon α-zingiberene synthase catalyzed the formation of α-zingiberene and other sesquiterpenes and monoterpenes in tomatoes (Davidovich-Rikanati et al., 2008). In our previous studies, we found that the overexpression of terpenoids and TPS genes, such as *SoLINS, SoNEOD, SoTPS6,SoSABS, SoCINS, SgGPS, SgFPPS*, and *SgLINS* from *S. officinalis* and *S. guaranitica* increased the accumulation of sesquiterpenes and monoterpenes in *N. tabacum* and *A. thaliana* plants (Ali et al., 2017, 2018). Finally, this property is found in our target gene, which catalyzes the formation of different types and amounts of terpenoids.

## Conclusion

We cloned a *SgCINS* gene that could encode a 1,8-cineole synthase and catalyze the biosynthesis of monoterpene from leaves of *S. guaranitica* plant. To examine the role of *SgCINS* in insect resistance, we transformed and expressed this gene into tobacco leaves. The availability of transgenic tobacco plants with overexpression of *SgCINS* made it possible to determine the biological function of *SgCINS*. In this study, we were particularly interested in the potential role of *SgCINS* in defense against cotton leafworms. At different times after the start of feeding (e.g., 0, 3, 6, 12, and 24 h), the RGR and the metabolic analysis were quantified. The RGR for cotton leafworms feeding on *SgCINS* overexpression tobacco was smaller than those on non-transgenic tobacco. In contrast, the levels of terpenoids accumulation from transgenic tobacco were larger than those from non-transgenic tobacco plants. Moreover, detecting high levels of terpenoids in transgenic tobacco might provide evidence that the various types of terpenoids are the principal compound that inhibits the growth and development of cotton leafworms. The results in this study contribute to our understanding the role of the *SgCINS* gene in tobacco defense against cotton leafworms. Most importantly, our study paves the way for engineering transgenic plants with enhanced insect resistance and reduces the application of pesticides.

## Data Availability Statement

The datasets presented in this study can be found in online repositories. The names of the repository/repositories and accession number(s) can be found below: https://www.ncbi.nlm.nih.gov/genbank/, KX893964.1.

## Author Contributions

MA conceived and designed the study. MA, DD, AA, NE, and DA performed the experiments. MA wrote the draft for the article. DD, AA, NE, and DA reviewed the final draft of the manuscript. All authors discussed the results and commented on the manuscript and participated in the analysis of the data, reading and, approving the final manuscript.

## Conflict of Interest

The authors declare that the research was conducted in the absence of any commercial or financial relationships that could be construed as a potential conflict of interest.

## Publisher’s Note

All claims expressed in this article are solely those of the authors and do not necessarily represent those of their affiliated organizations, or those of the publisher, the editors and the reviewers. Any product that may be evaluated in this article, or claim that may be made by its manufacturer, is not guaranteed or endorsed by the publisher.

## Footnotes

1
http://ipsort.hgc.jp/

2
http://web.expasy.org/protparam/

3
http://phylogeny.lirmm.fr/phylo_cgi/simple_phylogeny.cgi

4
https://www.ebi.ac.uk/Tools/msa/clustalo/

5
http://bar.utoronto.ca/efp/cgi-bin/efpWeb.cgi

6
https://www.arabidopsis.org/cgi-bin/Blast/TAIRblast.pl

7
http://bar.utoronto.ca/cell_efp/cgi-bin/cell_efp.cgi

8
http://www.idtdna.com/scitools/Applications/RealTimePCR/

9
http://www.sigmaaldrich.com

10
https://www.ebi.ac.uk/interpro/

11
http://www.ebi.ac.uk/interpro/
