# Supplementary material for: Cloning and Characterization of 1,8-Cineole Synthase (SgCINS) Gene From the Leaves of Salvia guaranitica Plant
Source: Front Plant Sci. 2022 Apr 15;13:869432. doi: 10.3389/fpls.2022.869432 (PMC9051517; doi:10.3389/fpls.2022.869432)
Supplement: Supplementary file 1 [file Table_1.doc]

Table S1. The major chemical composition and terpenes from transgenic *Nicotiana tabacum* leaves at different times from insect force-feeding assays*.*

| N | Compound name | R.T | Formula | M.W/Da | Terpene of component | *W.T* | | | | | *SgCINS* | | | | |
| --- | --- | --- | --- | --- | --- | --- | --- | --- | --- | --- | --- | --- | --- | --- | --- |
|  |  |  |  |  |  | 0 h | 3 h | 6 h | 12 h | 24 h | 0 h | 3 h | 6 h | 12 h | 24 h |
| 1 | m-Xylene; m-Dimethylbenzene | 5.217 | C8H10 | 106.165 |  | 0.04 | 0.2 |  |  |  |  |  |  |  | 0.04 |
| 2 | (8)Annulene | 5.966 | C8H8 | 104.1491 |  | 0.07 | 0.62 | 0.04 | 0.04 |  |  |  |  |  | 0.07 |
| 3 | n-Heptaldehyde; n-Heptanal; n-Heptylaldehyde | 6.354 | C7H14O | 114.1855 |  | 0.05 |  |  |  |  |  |  |  |  | 0.05 |
| 4 | Artificial Almond Oil | 9.524 | C7H6O | 106.1219 | organic |  | 0.38 |  |  |  |  |  |  |  |  |
| 5 | n-Decane | 11.999 | C10H22 | 142.2817 |  |  | 0.06 |  |  |  |  |  |  |  |  |
| 6 | 4-Pipecoline; γ-Pipecoline; 4-Methylpiperidine | 12.52 | C6H13N | 99.1741 |  |  |  |  |  |  | 0.01 |  |  |  |  |
| 7 | Benzenemethanol; α-Hydroxytoluene; α-Toluenol | 14.399 | C7H8O | 108.1378 |  |  | 0.04 |  |  |  |  |  |  |  |  |
| 8 | 2-Methylhexadecane | 15.499 | C17H36 | 240.4677 |  |  |  |  |  |  |  |  |  | 0.01 |  |
| 9 | n-Tridecane | 15.501 | C13H28 | 184.3614 |  |  |  |  |  |  |  | 0.02 |  |  |  |
| 10 | Undecane | 15.512 | C11H24 | 156.308 |  |  |  |  |  |  | 0.01 |  |  |  |  |
| 11 | 4,9-Di-n-propyldodecane | 15.519 | C18H38 | 254.4943 |  |  |  |  |  | 0.01 |  |  |  |  |  |
| 12 | Cyclohexanol, 2,6-dimethyl- | 16.306 | C8H16O | 128.212 |  |  |  |  |  | 0.01 | 0.01 | 0.01 |  | 0.01 |  |
| 13 | n-Dodecane; Adakane 12 | 17.653 | C12H26 | 170.3348 |  |  |  |  |  |  |  |  |  |  |  |
| 14 | n-Tridecane | 17.662 | C13H28 | 184.3614 |  |  | 0.52 |  |  |  |  |  |  |  |  |
| 15 | Undecane | 17.664 | C11H24 | 156.308 |  | 0.21 |  | 0.06 | 0.08 |  |  |  |  |  | 0.21 |
| 16 | β-Cyclocitral | 21.301 | C10H16O | 152.2334 | mono |  |  |  |  |  |  |  |  | 0.1 |  |
| 17 | 3,3-Dimethylhexane | 23.228 | C8H18 | 114.2285 |  |  |  |  |  |  |  |  |  | 0.01 |  |
| 18 | Dodecamethylcyclohexasiloxane | 24.061 | C12H36O6Si6 | 444.9236 |  |  |  |  |  | 0.06 | 0.02 | 0.04 | 0.04 | 0.3 |  |
| 19 | Capric ether | 25.033 | C20H42O | 298.5469 |  |  |  |  |  |  | 0.01 |  |  |  |  |
| 20 | Dodecamethylcyclohexasiloxane | 26.194 | C12H36O6Si6 | 444.9236 |  | 0.1 | 0.34 |  | 0.09 |  |  |  |  |  | 0.1 |
| 21 | trans-p-Menth-1-en-3-ol | 26.651 | C10H18O | 154.2493 | mono |  |  |  |  |  |  |  |  | 0.01 |  |
| 22 | Dodecandioic acid, bis(tert-butyldimethylsilyl) ester | 26.765 | C24H50O4Si2 | 458.8224 |  |  |  |  |  |  |  |  | 0.03 |  |  |
| 23 | L-(-)-Nicotine | 27.79 | C10H14N2 | 162.232 |  | 14.22 | 12.69 | 8.59 | 7.97 |  | 0.18 |  |  |  | 14.22 |
| 24 | Heptacosane | 27.949 | C27H56 | 380.7335 | alkane |  |  |  |  |  |  |  |  | 0.01 |  |
| 25 | n-Tetradecane | 27.963 | C14H30 | 198.388 |  |  |  |  |  | 0.02 | 0.01 |  |  |  |  |
| 26 | Tetradecamethylcycloheptasiloxane | 29.7 | C14H42O7Si7 | 519.0776 |  |  |  |  |  | 0.59 | 1.21 | 0.69 | 2.19 | 0.72 |  |
| 27 | n-Tetradecane | 29.742 | C14H30 | 198.388 |  |  | 0.04 |  |  |  |  |  |  |  |  |
| 28 | Methyl perillate | 30.187 | C11H16O2 | 180.2435 | mono |  |  |  |  | 0.09 |  | 0.05 | 0.03 | 0.03 |  |
| 29 | trans-β-Ionone | 30.704 | C13H20O | 192.2973 |  |  |  |  |  | 0.08 | 0.07 | 0.05 |  | 0.07 |  |
| 30 | Oxalic acid, bis(trimethylsilyl) ester | 30.897 | C8H18O4Si2 | 234.3971 |  |  |  |  |  |  |  |  | 0.05 |  |  |
| 31 | Topanol;Stavox | 31.447 | C15H24O | 220.3505 | sesqui |  |  |  |  | 3.33 | 0.08 | 1.15 | 0.7 | 0.61 |  |
| 32 | Phenol, 2-tert-butyl-4-methoxy- | 31.704 | C11H16O2 | 180.2435 |  |  | 0.24 |  |  |  |  |  |  |  |  |
| 33 | Tetradecamethylcycloheptasiloxane | 31.829 | C14H42O7Si7 | 519.0776 |  | 0.15 | 0.89 |  | 0.39 |  |  |  |  |  | 0.15 |
| 34 | (E)-β-Ionone | 32.308 | C13H20O | 192.2973 |  | 0.07 | 0.1 | 0.05 | 0.06 |  |  |  |  |  | 0.07 |
| 35 | Actinidiolide, dihydro- | 32.585 | C11H16O2 | 180.2435 | Mono |  |  |  |  | 0.03 | 0.05 | 0.02 | 0.01 | 0.03 |  |
| 36 | Topanol;Stavox | 33.083 | C15H24O | 220.3505 | Sesqui | 0.12 | 0.62 | 0.21 | 0.22 |  |  |  |  |  | 0.12 |
| 37 | Agidol 3 | 33.514 | C17H29NO | 263.4183 |  |  |  |  |  |  |  |  |  | 0.01 |  |
| 38 | Actinidiolide, dihydro- | 33.922 | C11H16O2 | 180.2435 |  | 0.03 | 0.08 |  |  |  |  |  |  |  | 0.03 |
| 39 | n-Hexadecane | 34.297 | C_16_H_34_ | 226.4412 |  |  |  |  |  | 0.02 |  |  |  |  |  |
| 40 | Hexadecamethylcyclooctasiloxane | 34.58 | C16H48O8Si8 | 593.2315 |  |  |  |  |  | 1.11 | 5.14 | 1.48 | 4.67 | 2.31 |  |

Table S1. Continued

| N | Compound name | R.T | Formula | M.W/Da | Terpene of component | *W.T* | | | | | *SgCINS* | | | | |
| --- | --- | --- | --- | --- | --- | --- | --- | --- | --- | --- | --- | --- | --- | --- | --- |
|  |  |  |  |  |  | 0 h | 3 h | 6 h | 12 h | 24 h | 0 h | 3 h | 6 h | 12 h | 24 h |
| 41 | (trans)-Carvyl propionate | 35.114 | C13H20O2 | 208.2967 | Mono |  |  |  |  |  |  |  |  | 0.02 |  |
| 42 | Cyclohexyl isopropylphosphonofluoridate | 35.327 | C9H18FO2P | 208.2102 |  |  |  |  | 0.04 |  |  |  |  |  |  |
| 43 | Heptyl propylphosphonofluoridate | 35.333 | C10H22FO2P | 224.253 |  | 0.12 |  |  |  |  |  |  |  |  |  |
| 44 | 1,4-Dimethyl-δ-3-tetrahydroacetophenone; 1,4-Dimethyl-3-cyclohexenyl methyl ketone; | 35.78 | C10H16O | 152.2334 | Mono |  |  |  |  |  | 0.04 | 0.07 | 0.04 | 0.03 | 0.03 |
| 45 | Menthofuran; Menthofurane;p-Mentha-3,8-diene, 3,9-epoxy- | 36.092 | C10H14O | 150.2176 | Mono |  |  |  |  |  |  |  |  | 0.04 |  |
| 46 | alpha.-Campholenal | 36.134 | C10H16O | 152.2334 | Mono |  |  |  |  |  |  | 0.04 |  |  |  |
| 47 | n-Cetane | 36.068 | C_16_H_34_ | 226.4412 |  |  |  | 0.06 |  |  |  | 0.12 |  |  |  |
| 48 | Hexadecamethylcyclooctasiloxane | 36.755 | C16H48O8Si8 | 593.2315 |  |  | 0.07 | 1.09 | 0.06 | 0.6 |  |  |  |  |  |
| 49 | Cetane | 37.149 | C_16_H_34_ | 226.4412 |  |  |  |  |  |  |  |  | 0.05 | 0.06 | 0.05 |
| 50 | Cetyl iodide; Hexadecyl iodide; | 37.18 | C16H33I | 352.3377 |  |  |  |  |  |  | 0.07 |  |  |  |  |
| 51 | 3-Methylphenyl Methylcarbamate | 37.323 | C9H11NO2 | 165.1891 |  | 0.1 |  |  |  |  |  |  |  |  |  |
| 52 | Myristaldehyde | 37.789 | C14H28O | 212.3715 |  |  |  |  |  |  |  | 0.17 | 0.23 | 0.22 |  |
| 53 | n-Tridecylaldehyde; Tridecanaldehyde; | 37.801 | C13H26O | 198.3449 |  |  |  |  |  |  | 0.13 |  |  |  | 0.08 |
| 54 | 3-Methyl-4-(1,3,3-trimethyl-7-oxa-bicyclo[4.1.0]hept-2-yl)-but-3-en-2-one | 38.59 | C14H22O2 | 222.323 |  | 0.13 |  |  | 0.05 | 0.06 |  |  |  |  |  |
| 55 | Germacra-1(10) | 38.609 | C15H20O2 | 232.3181 | sesqui |  | 0.05 |  |  |  |  |  |  |  |  |
| 56 | Octadeamethyl-cyclononasiloxane | 38.762 | C18H54O9Si9 | 667.3855 |  |  |  |  |  |  | 1.83 | 7.06 | 1.82 | 5.34 | 3.15 |
| 57 | Myristaldehyde | 39.401 | C14H28O | 212.3715 |  |  |  | 0.11 | 0.16 |  |  |  |  |  |  |
| 58 | Palmitaldehyde; 1-Hexadecanal; n-Hexadecanal | 39.403 | C16H32O | 240.4247 |  |  |  |  |  | 0.04 |  |  |  |  |  |
| 59 | Stearic acid; n-Octadecanoic acid | 39.711 | C18H36O2 | 284.4772 |  |  |  |  |  |  |  |  | 0.06 | 0.08 | 0.05 |
| 60 | n-Octadecane; Octadecan | 39.896 | C18H38 | 254.4943 |  |  |  |  |  |  |  | 0.07 |  |  |  |
| 61 | Cetane | 39.904 | C16H34 | 226.4412 |  |  |  |  |  |  | 0.08 |  |  |  |  |
| 62 | Hexadecamethylheptasiloxane | 40.702 | C16H48O6Si7 | 533.1472 |  |  |  |  |  |  |  | 0.06 |  |  |  |
| 63 | Myristic acid; n-Tetradecanoic acid | 40.723 | C14H28O2 | 228.3709 |  | 0.12 | 0.13 |  |  |  |  |  |  |  |  |
| 64 | Hexadecane, 1,2-epoxy-; Hexadecylene oxide | 40.811 | C16H32O | 240.4247 |  |  |  |  |  |  |  |  |  | 0.37 |  |
| 65 | octadecyne | 40.84 | C18H34 | 250.4626 |  |  |  |  |  |  |  | 0.68 |  |  |  |
| 66 | n-Pentadecanal | 40.847 | C15H30O | 226.3981 |  |  |  |  |  |  | 1.34 |  | 0.27 |  | 1.13 |
| 67 | Octadeamethyl-cyclononasiloxane | 40.983 | C18H54O9Si9 | 667.3855 |  |  |  | 1.27 |  | 0.65 |  |  |  |  |  |
| 68 | Adogen 73; Oleamide; Oleic acid amide | 41.474 | C18H35NO | 281.4766 |  |  |  |  |  |  |  |  | 0.06 |  |  |
| 69 | cis-9,cis-12-Octadecadienoic acid; cis,cis-Linoleic acid | 41.477 | C18H32O2 | 280.4455 |  |  |  |  |  |  |  |  |  | 0.05 | 0.06 |
| 70 | Phthalic acid, diisobutyl ester; Diisobutyl phthalate | 41.664 | C16H22O4 | 278.3435 |  |  |  |  |  |  |  | 0.07 |  |  | 0.05 |
| 71 | Citronellyl propionate | 41.942 | C13H24O2 | 212.3285 |  |  |  |  |  |  |  |  |  |  | 0.07 |
| 72 | Hexadecane, 1,2-epoxy-; Hexadecylene oxide; | 41.967 | C16H32O | 240.4247 |  |  |  |  |  |  | 0.06 |  |  |  |  |
| 73 | n-Pentadecanal | 41.962 | C15H30O | 226.3981 |  |  |  |  |  |  |  | 0.06 |  |  |  |
| 74 | 9,12-Octadecadien-1-ol, (Z,Z)- | 42.29 | C18H34O | 266.462 |  |  |  |  |  |  |  |  | 0.15 |  |  |
| 75 | 1-Nonadecanol; Nonadecanol; | 42.279 | C19H40O | 284.5203 |  |  |  |  |  |  |  |  |  |  | 0.1 |
| 76 | cis-9,cis-12-Octadecadienoic acid; cis,cis-Linoleic acid | 42.291 | C18H32O2 | 280.4455 |  |  |  |  |  |  |  |  |  | 0.13 |  |
| 77 | Hexadecamethylheptasiloxane | 42.466 | C16H48O6Si7 | 533.1472 |  |  |  |  |  |  | 2.53 |  |  |  |  |
| 78 | Octadeamethyl-cyclononasiloxane | 42.468 | C18H54O9Si9 | 667.3855 |  |  |  |  |  |  |  | 7.02 | 3.29 | 5.83 | 3.54 |
| 79 | n-Pentadecanal | 42.613 | C15H30O | 226.3981 |  | 0.31 |  | 0.37 | 0.26 |  |  |  |  |  |  |
| 80 | Hexadecane, 1,2-epoxy-; Hexadecylene oxide; | 42.615 | C16H32O | 240.4247 |  |  |  |  |  | 0.17 |  |  |  |  |  |

Table S1. Continued

| N | Compound name | R.T | Formula | M.W/Da | Terpene of component | *W.T* | | | | | *SgCINS* | | | | |
| --- | --- | --- | --- | --- | --- | --- | --- | --- | --- | --- | --- | --- | --- | --- | --- |
|  |  |  |  |  |  | 0 h | 3 h | 6 h | 12 h | 24 h | 0 h | 3 h | 6 h | 12 h | 24 h |
| 81 | 1,2-Epoxyhexadecane | 42.625 | C16H32O | 240.4247 |  |  | 0.18 |  |  |  |  |  |  |  |  |
| 82 | Phthalic acid, diisobutyl ester; Diisobutyl phthalate | 43.218 | C16H22O4 | 278.3435 |  | 0.09 |  |  |  |  |  |  |  |  |  |
| 83 | Palmitic acid, methyl ester | 43.248 | C17H34O2 | 270.4507 | ester |  |  |  |  |  | 1.12 | 0.14 | 0.09 | 0.08 | 0.14 |
| 84 | Linolenic acid; α-Linolenic acid; | 44.086 | C18H30O2 | 278.4296 |  | 0.44 | 0.46 | 0.16 | 0.42 | 0.26 |  |  |  |  |  |
| 85 | Linolenic acid, methyl ester | 44.131 | C19H32O2 | 292.4562 | ester |  |  |  |  |  | 1.51 | 1.22 | 1.88 | 1.7 | 1.34 |
| 86 | Linolenic acid; α-Linolenic acid; | 44.155 | C18H30O2 | 278.4296 |  | 0.2 | 0.22 |  |  |  |  |  |  |  |  |
| 87 | n-Cetane; n-Hexadecane; Cetane | 44.284 | C_16_H_34_ | 226.4412 |  |  |  |  |  | 0.11 |  |  |  |  |  |
| 88 | Hexadecamethylheptasiloxane | 44.737 | C16H48O6Si7 | 533.1472 |  |  |  |  |  | 0.61 |  | 0.26 |  |  |  |
| 89 | Octadeamethyl-cyclononasiloxane | 44.739 | C18H54O9Si9 | 667.3855 |  |  |  | 2.69 |  |  |  |  |  |  |  |
| 90 | n-Hexadecanoic acid | 44.777 | C16H32O2 | 256.4241 |  |  |  |  |  |  | 6.5 | 7.47 | 6.13 | 6.06 | 6 |
| 91 | Cetylic acid | 44.922 | C17H34O2 | 270.4507 | ester |  | 0.15 | 0.16 | 0.19 |  |  |  |  |  |  |
| 92 | 9,12-Octadecadien-1-ol, (Z,Z)- | 45.123 | C18H34O | 266.462 |  | 0.26 |  |  | 0.25 |  |  |  |  |  |  |
| 93 | cis-9,cis-12-Octadecadienoic acid; cis,cis-Linoleic acid | 45.135 | C18H32O2 | 280.4455 |  |  | 0.15 |  |  | 0.34 |  |  |  |  |  |
| 94 | γ-Linolenic acid | 45.265 | C18H30O2 | 278.4296 |  | 2.31 | 1.16 |  | 2.61 | 3.32 |  |  |  |  |  |
| 95 | trans-Oleic acid; trans-9-Octadecenoic acid; Elaidic acid | 45.365 | C18H34O2 | 282.4614 |  | 0.98 | 0.65 |  |  |  |  |  |  |  |  |
| 96 | Erucic acid;δ13-cis-Docosenoic acid; cis-13-Docosenoic acid; | 45.374 | C22H42O2 | 338.5677 |  |  |  |  | 1.08 |  |  |  |  |  |  |
| 97 | trans-Retinyl acetate | 45.497 | C22H32O2 | 328.4883 |  | 1.46 | 1.77 | 1.74 | 2.89 | 1.18 |  |  |  |  |  |
| 98 | Margaric acid methyl ester; Methyl heptadecanoate; Methyl margarat | 45.705 | C18H36O2 | 284.4772 |  |  |  |  |  |  | 0.56 |  |  |  | 0.06 |
| 99 | Hexadecamethylcyclooctasiloxane | 45.851 | C16H48O8Si8 | 593.2315 |  |  |  |  |  |  | 3.19 | 7.59 | 2.81 | 5.78 | 3.94 |
| 100 | Palmitic acid | 45.96 | C16H32O2 | 256.4241 |  | 15.28 | 6.84 | 5.36 | 13.1 | 13.86 |  |  |  |  |  |
| 101 | (+)-Ledol | 46 | C15H26O | 222.3663 | sesqui |  |  |  |  |  |  |  | 0.6 |  |  |
| 102 | 4,8,13-Duvatriene-1,3-Diol | 46.045 | C_20_H_34_O_2_ | 306.4828 | diter |  |  |  |  |  | 0.32 | 0.15 |  | 0.22 | 0.45 |
| 103 | Retinol, acetate, all-trans- | 46.156 | C22H32O2 | 328.4883 |  | 1.1 | 0.85 | 0.72 | 1.33 | 1 |  |  |  |  |  |
| 104 | (Z)-7-Hexadecenal | 46.335 | C16H30O | 238.4088 |  |  |  |  |  |  |  |  |  |  | 0.12 |
| 105 | Pentadecylic acid; n-Pentadecanoic acid | 46.352 | C15H30O2 | 242.3975 |  |  |  |  |  |  | 0.1 |  | 0.16 | 0.13 |  |
| 106 | Retinyl acetate | 46.365 | C22H32O2 | 328.4883 |  | 1.61 | 1.94 | 1.76 | 2.86 | 1.46 |  |  |  |  |  |
| 107 | Stearic acid | 46.35 | C18H36 | 284.477 |  |  |  |  |  |  |  | 0.11 |  |  |  |
| 108 | Cholest-5-en-3-ol (3.beta.)-, carbonochloridate | 46.442 | C28H45ClO2 | 449.1087 |  |  |  |  |  |  |  |  | 0.56 |  |  |
| 109 | δ-Guajene | 46.494 | C15H24 | 204.3511 | sesqui |  |  |  |  |  | 0.36 |  |  | 0.29 | 0.47 |
| 110 | Cycloartanyl acetate | 46.514 | C32H54O2 | 470.77 |  |  |  |  |  |  |  | 0.24 |  |  |  |
| 111 | n-Heneicosane; n-henicosane; henicosane | 46.769 | C21H44 | 296.5741 |  |  |  | 0.13 |  | 0.18 |  |  |  |  |  |
| 112 | geranylgeraniol | 46.761 | C22H36O2 | 332.52 | diter |  |  |  |  |  |  | 0.13 |  |  | 0.51 |
| 113 | d-Ledol | 46.804 | C15H26O | 222.3663 | sesqui |  |  |  |  |  | 0.4 |  | 0.58 | 0.3 |  |
| 114 | all-trans-Vitamin A acetate | 47.018 | C22H32O2 | 328.4883 |  |  |  |  |  |  | 0.37 |  | 0.7 | 0.31 | 0.57 |
| 115 | Linoleic acid, methyl ester; Methyl cis,cis-9,12-octadecadienoate | 47.264 | C19H34O2 | 294.4721 |  |  |  |  |  |  | 4.51 | 0.13 | 0.4 | 0.22 | 0.66 |
| 116 | Linolenic acid, methyl ester | 47.404 | C19H32O2 | 292.4562 | ester |  |  |  |  |  |  | 0.27 | 0.72 | 0.41 | 0.77 |
| 117 | Linoleic; Linoleic acid; Linolic acid | 47.42 | C18H32O2 | 280.4455 |  |  |  |  |  |  | 5.29 |  |  |  |  |
| 118 | Cycloartanyl acetate | 47.514 | C32H54O2 | 470.77 |  |  |  |  |  |  |  | 0.1 |  |  |  |
| 119 | Methyl elaidate | 47.549 | C19H36O2 | 296.4879 |  |  |  |  |  |  | 0.44 |  |  |  |  |
| 120 | Stearic acid; n-Octadecanoic acid | 47.605 | C18H36O2 | 284.4772 |  |  |  |  | 0.34 | 0.11 |  |  |  |  |  |

Table S1. Continued

| N | Compound name | R.T | Formula | M.W/Da | Terpene of component | *W.T* | | | | | *SgCINS* | | | | |
| --- | --- | --- | --- | --- | --- | --- | --- | --- | --- | --- | --- | --- | --- | --- | --- |
|  |  |  |  |  |  | 0 h | 3 h | 6 h | 12 h | 24 h | 0 h | 3 h | 6 h | 12 h | 24 h |
| 121 | Phytol | 47.7 | C20H40O | 296.531 | diter |  |  |  |  |  | 2.63 | 1.81 | 4.66 | 2.94 | 2.78 |
| 122 | Globulol | 47.795 | C15H26O | 222.3663 | Sesgui |  |  | 0.22 | 0.45 | 0.13 |  |  |  |  |  |
| 123 | 4,8,13-Duvatriene-1,3-Diol | 47.804 | C20H34O2 | 306.4828 | diter |  | 0.17 |  |  |  |  |  |  |  |  |
| 124 | Crystalets | 47.888 | C22H32O2 | 328.4883 |  | 0.84 |  | 1.04 | 1.66 | 0.59 |  |  |  |  |  |
| 125 | all-trans-Retinol acetate | 47.903 | C22H32O2 | 328.4883 |  |  | 1.11 |  |  |  |  |  |  |  |  |
| 126 | All-trans-Geranylgeraniol | 47.998 | C22H36O2 | 332.52 | diter | 0.27 |  | 0.4 | 0.51 | 0.17 |  |  |  |  |  |
| 127 | β-Elemol | 48.015 | C15H26O | 222.3663 | sesqui |  | 0.34 |  |  |  |  |  |  |  |  |
| 128 | Methyl stearate | 48.058 | C19H38O2 | 298.5038 |  |  |  |  |  |  | 0.83 |  |  |  |  |
| 129 | 4,8,13-Duvatriene-1,3-Diol | 48.126 | C20H34O2 | 306.4828 | diter |  |  |  |  |  |  |  | 1.06 | 0.36 | 0.92 |
| 130 | geranylgeraniol | 48.149 | C22H36O2 | 332.52 | diter | 3.65 |  |  | 7.19 |  |  |  |  |  |  |
| 131 | Hexadecamethylcyclooctasiloxane | 48.169 | C16H48O8Si8 | 593.2315 |  |  |  | 9.59 |  |  |  |  |  |  |  |
| 132 | (-)-Globulol | 48.169 | C15H26O | 222.3663 | sesqui |  | 4.99 |  |  | 3.35 |  |  |  |  |  |
| 133 | 4,8,13-Duvatriene-1,3-Diol | 48.181 | C_20_H_34_O_2_ | 306.4828 | diter |  |  |  |  |  | 0.65 | 0.08 |  |  |  |
| 134 | [(2-Isopropyl-5-methylcyclohexyl)oxy](trimethyl)silane | 48.371 | C13H28OSi | 228.446 |  |  |  |  |  |  |  |  | 0.54 |  |  |
| 135 | Propane, 1,2-dibromo-3-chloro- | 48.404 | C3H5Br2Cl | 236.333 |  |  |  |  |  |  | 0.56 | 0.16 |  | 0.25 | 0.62 |
| 136 | geranylgeraniol | 48.404 | C22H36O2 | 332.52 | diter | 4.22 | 5.58 | 6.02 | 8.64 | 3.42 |  |  | 0.55 | 0.17 | 0.48 |
| 137 | Widdrol | 48.477 | C15H26O | 222.3663 | sesqui | 1.33 |  |  | 2.79 |  |  |  |  |  |  |
| 138 | (4aS,7S)-1,1,4a,7-Tetramethyl-2,3,4,4a,5,6,7,8-octahydro-1H-benzo[7]annulen-7-ol | 48.482 | C15H26O | 222.366 | Sesqui |  |  |  |  | 1.05 |  |  |  |  |  |
| 139 | steviol | 48.495 | C20H30O3 | 318.45 | diter |  | 1.72 | 1.82 |  |  |  |  |  |  |  |
| 140 | cis-Caryophyllene epoxide; trans-caryophyllene oxide | 48.608 | C15H24O | 220.3505 | sesqui | 0.2 |  |  |  |  |  |  |  |  |  |
| 141 | 4,8,13-Duvatriene-1,3-Diol | 48.618 | C20H34O2 | 306.4828 | diter |  |  |  |  | 0.2 |  |  |  |  |  |
| 142 | taraxastane | 48.63 | C30H50 | 410.718 | triter |  | 0.27 | 0.39 | 0.67 |  |  |  |  |  |  |
| 143 | trans-Vitamin A acetate | 48.705 | C22H32O2 | 328.4883 |  | 1.28 | 1.78 | 1.79 | 2.91 | 1.13 |  |  |  |  |  |
| 144 | Octadeamethyl-cyclononasiloxane | 48.918 | C18H54O9Si9 | 667.3855 |  |  |  |  |  |  |  |  | 10.27 | 13.74 | 10.78 |
| 145 | Linolenic acid | 48.938 | C18H30O2 | 278.4296 |  |  |  |  |  |  | 13.31 | 14.71 | 11.27 |  | 10.2 |
| 146 | Linolenic acid, methyl ester | 49.069 | C19H32O2 | 292.4562 | ester |  |  |  | 0.48 |  |  |  |  |  |  |
| 147 | Linolenic acid, ethyl ester;Ethyl linolenate; Ethyl α-linolenate | 49.087 | C20H34O2 | 306.4828 | ester |  | 0.66 |  |  |  |  |  |  |  |  |
| 148 | Linolenic acid, methyl ester | 49.069 | C19H32O2 | 292.4562 | ester | 0.48 |  | 0.36 |  |  |  |  |  |  |  |
| 149 | n-Heneicosane; n-henicosane; henicosane | 49.154 | C21H44 | 296.5741 |  |  | 2.3 |  |  | 4.69 |  |  |  |  |  |
| 150 | g-Elemene | 49.284 | C15H24 | 204.351 | sesqui | 3.82 |  |  |  | 2.98 |  |  |  |  |  |
| 151 | α-4,8,13-Duvatriene-1,3-Diol | 49.3 | C20H34O2 | 306.4828 | diter |  | 4.94 | 4.94 | 7.26 |  |  |  |  |  |  |
| 152 | Stearic acid | 49.321 | C18H36 | 284.477 |  |  |  |  |  |  |  | 1.5 | 2.22 | 1.99 | 1.85 |
| 153 | Phytol | 49.331 | C20H40O | 296.531 | diter | 5.2 | 3.86 |  |  | 3.65 |  |  |  |  |  |
| 154 | Stearic acid; n-Octadecanoic acid | 49.356 | C18H36O2 | 284.4772 |  |  |  |  |  |  | 1.63 |  |  |  |  |
| 155 | Cycloartanyl acetate | 49.612 | C32H54O2 | 470.77 |  |  |  |  |  |  |  |  |  | 0.42 |  |
| 156 | (-)-Ledol | 49.631 | C15H26O | 222.3663 | sesqui |  |  |  |  |  |  |  |  |  | 0.68 |
| 157 | n-Octadecyl chloride; Octadecyl chloride | 49.631 | C18H37Cl | 288.939 |  |  |  |  |  |  | 0.58 |  |  |  |  |
| 158 | Viridiflorine; (+)-Ledene | 49.841 | C15H24 | 204.3511 | sesqui |  |  |  |  | 1.44 |  |  | 0.66 |  |  |
| 159 | β-4,8,13-Duvatriene-1,3-Diol | 49.836 | C20H34O2 | 306.4828 | diter | 1.84 | 2.27 | 1.97 | 3.65 |  |  |  |  |  |  |
| 160 | Octadecanol acetate; Octadecyl acetate; n-Octadecyl acetate; Stearyl acetate | 49.917 | C20H40O2 | 312.5304 |  |  |  |  |  |  | 0.24 |  |  |  |  |

Table S1. Continued

| N | Compound name | R.T | Formula | M.W/Da | Terpene of component | *W.T* | | | | | *SgCINS* | | | | |
| --- | --- | --- | --- | --- | --- | --- | --- | --- | --- | --- | --- | --- | --- | --- | --- |
|  |  |  |  |  |  | 0 h | 3 h | 6 h | 12 h | 24 h | 0 h | 3 h | 6 h | 12 h | 24 h |
| 161 | α-Linolenic acid; | 50.161 | C18H30O2 | 278.4296 |  | 29.44 | 16.74 | 2.46 | 20.82 | 26.47 |  |  |  |  |  |
| 162 | Cedrol | 50.227 | C15H26O | 222.3663 | sesqui |  |  |  |  |  |  | 0.07 |  |  |  |
| 163 | Labda-8(17),14-dien-6,13-diol | 50.411 | C_20_H_34_O_2_ | 306.4828 | diter |  |  |  |  |  |  | 1.49 |  |  |  |
| 164 | Ledol | 50.518 | C15H26O | 222.3663 | sesqui |  |  |  |  |  | 13.02 |  | 22.49 | 14.42 | 20.61 |
| 165 | Stearic acid; n-Octadecanoic acid | 50.656 | C18H36O2 | 284.4772 |  | 1.75 | 0.8 | 0.4 | 1.54 | 1.97 |  |  |  |  |  |
| 166 | Hexadecamethylheptasiloxane | 50.843 | C16H48O6Si7 | 533.1472 |  |  |  |  |  |  | 0.05 | 0.12 |  |  |  |
| 167 | Isopropyl linoleate | 51.055 | C21H38O2 | 322.5252 |  |  |  |  |  |  |  |  |  |  | 0.02 |
| 168 | α-Elemol; β-Elemol | 51.059 | C15H26O | 222.3663 | sesqui | 0.3 |  |  |  | 0.24 |  |  |  |  |  |
| 169 | Epiglobulol | 51.086 | C15H26O | 222.3663 | sesqui |  | 0.35 | 0.28 | 0.55 |  |  |  |  |  |  |
| 170 | trans-Lycopene | 51.066 | C40H56 | 536.8726 | tetraterpene |  |  |  |  |  |  | 0.33 |  |  |  |
| 171 | (+)-Ledol; d-Ledol | 51.314 | C15H26O | 222.3663 | Sesqui |  |  |  | 0.2 |  |  |  |  |  |  |
| 172 | Octadeamethyl-cyclononasiloxane | 51.403 | C18H54O9Si9 | 667.3855 |  |  |  | 6.87 |  | 0.63 |  |  |  |  |  |
| 173 | Isoabienol | 51.551 | C22H36O2 | 332.52 | diter |  |  |  |  |  |  |  | 0.06 |  |  |
| 174 | henicosane | 51.552 | C21H44 | 296.5741 |  |  | 0.21 | 0.21 |  | 0.14 |  |  |  |  |  |
| 175 | Cycloartanyl acetate | 51.567 | C32H54O2 | 470.77 |  |  |  |  |  |  |  |  |  |  | 0.05 |
| 176 | Octadeamethyl-cyclononasiloxane | 52.007 | C18H54O9Si9 | 667.3855 |  |  |  |  |  |  | 5.16 | 8.85 | 4.28 | 6.86 | 5.09 |
| 177 | cis-11-Eicosenoic acid, methyl ester | 52.131 | C21H40O2 | 324.5411 |  |  |  |  |  |  | 2.64 |  |  |  |  |
| 178 | 4,8,13-Duvatriene-1,3-Diol | 52.219 | C20H34O2 | 306.4828 | diter | 0.92 |  |  |  |  |  |  |  |  |  |
| 179 | (+)-Ledol; d-Ledol | 52.23 | C15H26O | 222.3663 | Sesgui |  |  | 3.09 | 5.35 | 0.97 |  |  |  |  |  |
| 180 | Sclareol | 52.251 | C20H34O2 | 306.4828 | diter |  | 2.78 |  |  |  |  |  |  |  |  |
| 181 | cis-11-Eicosenoic acid, methyl ester | 52.313 | C21H40O2 | 324.5411 |  |  |  |  |  |  | 0.24 |  |  |  |  |
| 182 | cis-Lycopene | 52.395 | C40H56 | 536.8726 | tetraterpene |  |  |  |  |  |  | 0.37 |  |  |  |
| 183 | Methyl arachisate | 52.901 | C21H42O2 | 326.557 |  |  |  |  |  |  | 0.3 |  |  |  |  |
| 184 | Ascaridole | 53.511 | C10H16O2 | 168.2328 | Mono |  |  |  |  |  |  | 0.11 |  |  |  |
| 185 | Tetrapentacontane | 53.794 | C54H110 | 759.4512 |  |  | 0.7 |  |  |  |  |  |  |  |  |
| 186 | epi-α-Elemol | 54.143 | C15H26O | 222.3663 | sesqui |  |  |  |  |  |  |  | 0.4 |  |  |
| 187 | Hexadecan-1-ol | 54.149 | C16H34O | 242.441 |  |  |  |  | 0.09 |  |  |  |  |  |  |
| 188 | Docosanol;Behenyl alcohol; n-Docosanol; Abreva | 54.172 | C22H46O | 326.6 |  |  |  |  |  | 0.17 |  |  |  |  |  |
| 189 | 7,10-Hexadecadienoic acid, methyl ester | 54.188 | C17H30O2 | 266.4189 |  |  |  |  |  |  |  | 0.09 |  |  |  |
| 190 | Squalene | 54.215 | C30H50 | 410.718 | triter |  |  |  |  |  | 0.18 |  |  | 0.21 | 0.36 |
| 191 | Nonadec-1-ene | 54.224 | C19H38 | 266.505 |  |  | 0.77 |  |  |  |  |  |  |  |  |
| 192 | n-Heneicosane; n-henicosane; henicosane | 54.566 | C21H44 | 296.5741 |  |  | 4.75 | 0.2 |  | 1.17 |  |  |  |  |  |
| 193 | 4,8,13-Duvatriene-1,3-Diol | 54.733 | C_20_H_34_O_2_ | 306.4828 | diter |  |  |  |  |  |  | 0.13 |  |  |  |
| 194 | Hexadecamethylheptasiloxane | 54.929 | C16H48O6Si7 | 533.1472 |  |  |  |  |  |  | 0.06 | 0.09 |  |  |  |
| 195 | trans-2-undecenoic acid | 55.016 | C11H20O2 | 184.2753 |  |  |  |  |  |  |  |  |  | 0.1 |  |
| 196 | n-Heneicosane; n-henicosane; henicosane | 55.079 | C21H44 | 296.5741 |  |  |  |  |  |  | 0.28 |  |  |  |  |
| 197 | Amylethylmethylcarbinol; 3-Methyl-3-octanol; | 55.227 | C9H20O | 144.2545 |  |  |  |  |  |  |  |  |  | 0.17 |  |
| 198 | 4,8,13-Duvatriene-1,3-Diol | 55.529 | C_20_H_34_O_2_ | 306.4828 | diter |  |  |  |  |  |  | 0.12 |  |  |  |
| 199 | Tetrapentacontane | 55.543 | C54H110 | 759.4512 |  |  | 0.62 |  |  |  |  |  |  |  |  |
| 200 | n-Hexadecyl ethanoate | 55.554 | C18H36O2 | 284.4772 |  |  |  |  |  |  | 0.06 |  |  |  |  |

Table S1. Continued

| N | Compound name | R.T | Formula | M.W/Da | Terpene of component | *W.T* | | | | | *SgCINS* | | | | |
| --- | --- | --- | --- | --- | --- | --- | --- | --- | --- | --- | --- | --- | --- | --- | --- |
|  |  |  |  |  |  | 0 h | 3 h | 6 h | 12 h | 24 h | 0 h | 3 h | 6 h | 12 h | 24 h |
| 201 | Octadeamethyl-cyclononasiloxane | 55.632 | C18H54O9Si9 | 667.3855 |  |  |  | 6.1 |  | 0.43 |  |  |  |  |  |
| 202 | Octadeamethyl-cyclononasiloxane | 56.308 | C18H54O9Si9 | 667.3855 |  |  |  |  |  |  | 4.8 | 8.03 | 4.19 | 6.21 | 4.73 |
| 203 | Adogen 73; Oleamide; Oleic acid amide | 56.669 | C18H35NO | 281.4766 |  | 0.4 |  |  |  | 0.21 |  |  |  |  |  |
| 204 | 6,9-Octadecadienoic acid, methyl ester | 56.724 | C19H34O2 | 294.4721 |  |  | 0.28 |  | 0.22 |  |  |  |  |  |  |
| 205 | 10-epi-Elemol | 56.729 | C15H26O | 222.3663 | sesqui |  |  | 0.19 |  |  |  |  |  |  |  |
| 206 | Linolenic acid; α-Linolenic acid; | 57.44 | C18H30O2 | 278.4296 |  |  |  |  |  |  |  |  | 0.04 |  |  |
| 207 | Dihomo-γ-linolenic acid | 57.445 | C20H34O2 | 306.4828 |  |  |  |  |  |  |  |  |  | 0.02 |  |
| 208 | (Z)-7-Hexadecenal | 57.452 | C16H30O | 238.4088 |  |  |  |  |  |  |  |  |  |  | 0.02 |
| 209 | Myristamide; Myristic acid amide; Myristic amide | 57.801 | C14H29NO | 227.3862 |  | 0.07 |  |  |  |  |  |  |  |  |  |
| 210 | Palmitamide; n-Hexadecanamide; Amide HPL | 57.804 | C16H33NO | 255.4393 |  |  |  |  |  | 0.04 |  |  |  |  |  |
| 211 | Nonadec-1-ene | 58.254 | C19H38 | 266.505 |  | 0.15 |  |  |  |  |  |  |  |  |  |
| 212 | n-Tetracontane | 58.51 | C40H82 | 563.0791 |  |  |  | 0.26 |  |  |  |  |  |  |  |
| 213 | n-Heneicosane; n-henicosane; henicosane | 58.528 | C21H44 | 296.5741 |  |  | 0.26 |  |  | 0.09 |  |  |  |  |  |
| 214 | 2-Hexyl-1-decanol; 2-Hexyldecanol | 59.047 | C16H34O | 242.4406 |  |  |  |  |  |  |  |  | 0.41 | 0.31 | 0.38 |
| 215 | n-Pentatriacontane; n-Pentatriacotane | 59.159 | C35H72 | 492.9462 |  |  |  |  |  |  | 0.42 | 0.3 |  |  |  |
| 216 | Methyl cis-13-docosenoate | 59.402 | C23H44O2 | 352.5943 |  |  |  |  |  |  | 0.19 |  |  |  |  |
| 217 | Naphthalene, 2,2'-oxybis-; di-2-naphthyl ether | 59.797 | C20H14O | 270.3246 |  |  |  |  |  |  |  |  |  |  | 0.01 |
| 218 | Stearaldehyde; Octadecyl aldehyde | 59.903 | C18H36O | 268.4778 |  |  | 0.2 |  |  | 0.54 |  |  |  |  |  |
| 219 | Phthalic acid dioctyl ester; | 60.682 | C_24_H_38_O_4_ | 390.5561 |  |  |  |  |  |  | 0.37 | 0.5 | 0.27 | 0.27 | 0.23 |
| 220 | Hexadecamethylheptasiloxane | 61.153 | C16H48O6Si7 | 533.1472 |  |  |  |  |  |  | 0.05 |  |  |  |  |
| 221 | Octadeamethyl-cyclononasiloxane | 61.89 | C18H54O9Si9 | 667.3855 |  |  |  | 5.7 |  | 0.38 |  |  |  |  |  |
| 222 | Linolenic acid, ethyl ester;Ethyl linolenate; Ethyl α-linolenate | 61.85 | C20H34O2 | 306.4828 | ester |  |  |  |  |  |  |  | 0.03 |  |  |
| 223 | Linolenic acid, methyl ester | 61.956 | C19H32O2 | 292.4562 | ester |  |  |  |  |  | 0.03 |  |  |  | 0.03 |
| 224 | Octadeamethyl-cyclononasiloxane | 62.62 | C18H54O9Si9 | 667.3855 |  |  |  |  |  |  | 4.57 | 7.21 | 3.91 | 5.52 | 4.24 |
| 225 | Docosanol;Behenyl alcohol; n-Docosanol; Abreva | 63.128 | C22H46O | 326.6 |  | 0.07 |  | 0.05 | 0.08 | 0.68 |  |  |  |  |  |
| 226 | Nonadec-1-ene | 63.133 | C19H38 | 266.505 |  |  | 0.53 |  |  |  |  |  |  |  |  |
| 227 | n-Cetane; n-Hexadecane; Cetane | 63.566 | C_16_H_34_ | 226.4412 |  |  |  |  | 0.04 |  |  |  |  |  |  |
| 228 | n-Tetracontane | 63.599 | C40H82 | 563.0791 |  |  |  |  |  | 1.33 |  |  |  |  |  |
| 229 | n-Heneicosane; n-henicosane; henicosane | 63.633 | C21H44 | 296.5741 |  |  | 3.08 | 0.23 |  |  |  |  |  |  |  |
| 230 | n-Nonacosane | 64.181 | C29H60 | 408.7867 | alkane |  |  |  |  |  |  |  | 0.07 |  |  |
| 231 | n-Tetracontane | 64.294 | C40H82 | 563.0791 |  |  |  |  |  |  | 0.21 |  |  | 0.07 | 0.07 |
| 232 | n-henicosane; henicosane | 64.285 | C21H44 | 296.5741 |  |  |  |  |  |  |  | 0.09 |  |  |  |
| 233 | Phthalic acid dioctyl ester; | 64.84 | C_24_H_38_O_4_ | 390.5561 |  | 0.17 | 0.27 | 0.1 | 0.06 | 0.2 |  |  |  |  |  |
| 234 | Hexacosyl acetate | 64.929 | C28H56O2 | 424.743 |  |  |  |  |  |  |  |  | 0.02 |  |  |
| 235 | 1,2-Epoxyoctadecane; Hexadecyl oxirane | 65.244 | C18H36O | 268.4778 |  |  |  |  |  | 0.1 |  |  |  |  |  |
| 236 | Linolenic acid, ethyl ester;Ethyl linolenate; Ethyl α-linolenate | 66.362 | C20H34O2 | 306.4828 | ester |  |  |  | 0.03 |  |  |  |  |  |  |
| 237 | Octadeamethyl-cyclononasiloxane | 67.014 | C18H54O9Si9 | 667.3855 |  |  |  |  |  |  |  |  | 0.03 |  | 0.04 |
| 238 | allyl valerate | 68.738 | C8H14O2 | 142.1956 |  |  |  |  |  |  |  |  | 0.05 |  |  |
| 239 | n-Octacosanol; Cluytyl alcohol; Montanyl alcohol | 68.743 | C28H58O | 410.7595 |  |  | 0.12 |  |  |  |  |  |  |  |  |
| 240 | Isovaleric anhydride; iso-Pentanoic anhydride | 68.826 | C10H18O3 | 186.2481 | Mono |  |  |  |  |  |  |  |  |  | 0.04 |

Table S1. Continued

| N | Compound name | R.T | Formula | M.W/Da | Terpene of component | *W.T* | | | | | *SgCINS* | | | | |
| --- | --- | --- | --- | --- | --- | --- | --- | --- | --- | --- | --- | --- | --- | --- | --- |
|  |  |  |  |  |  | 0 h | 3 h | 6 h | 12 h | 24 h | 0 h | 3 h | 6 h | 12 h | 24 h |
| 241 | Mandelic acid di(tert-butyldimethylsilyl)- | 68.859 | C20H36O3Si2 | 380.669 |  |  |  |  |  |  |  |  |  | 0.04 |  |
| 242 | Nonadec-1-ene | 68.916 | C19H38 | 266.505 |  | 0.15 |  |  |  |  |  |  |  |  |  |
| 243 | Hexadecamethylheptasiloxane | 68.92 | C16H48O6Si7 | 533.1472 |  |  |  |  |  |  | 0.06 | 0.07 |  |  |  |
| 244 | n-Heneicosane; n-henicosane; henicosane | 69.231 | C21H44 | 296.5741 |  |  | 0.27 |  |  | 0.21 |  |  |  |  |  |
| 245 | Octadeamethyl-cyclononasiloxane | 69.519 | C18H54O9Si9 | 667.3855 |  |  |  | 6.13 |  | 0.37 |  |  |  |  |  |
| 246 | Octadeamethyl-cyclononasiloxane | 69.966 | C18H54O9Si9 | 667.3855 |  |  |  |  |  |  | 5.02 | 6.84 | 3.97 | 5.21 | 4.11 |
| 247 | Eicosane, 1-cyclohexyl- | 70.445 | C26H52 | 364.6911 |  |  | 0.15 |  |  |  |  |  |  |  |  |
| 248 | Stearaldehyde; Octadecyl aldehyde | 71.074 | C18H36O | 268.4778 |  |  | 0.22 |  |  | 0.29 |  |  |  |  |  |
| 249 | 2-Hexyl-1-decanol; 2-Hexyldecanol | 71.523 | C16H34O | 242.4406 |  |  |  |  |  |  |  |  |  |  | 0.01 |
| 250 | Tetrapentacontane | 72.012 | C54H110 | 759.4512 |  |  | 0.18 |  |  |  |  |  |  | 0.02 |  |
| 251 | 3-Ethyl-5-(2'-ethylbutyl)octadecane | 72.51 | C26H54 | 366.707 |  |  | 0.3 |  |  |  |  |  |  | 0.2 |  |
| 252 | n-Tetracosane | 72.57 | C24H50 | 338.6538 |  |  |  |  |  |  |  |  |  |  | 0.02 |
| 253 | n-Dotriacontane; Bicetyl | 72.657 | C32H66 | 450.8664 |  |  |  |  |  |  | 0.01 |  |  |  |  |
| 254 | (-)-Myrtenol | 73.15 | C10H16O | 152.2334 | mono |  |  |  |  |  |  |  | 0.04 |  |  |
| 255 | Benzene, [[(1-ethenyl-1,5-dimethyl-4-hexenyl)oxy]methyl]- | 73.171 | C17H24O | 244.37 |  |  |  |  |  |  |  |  |  | 0.04 |  |
| 256 | Tetrapentacontane | 73.497 | C54H110 | 759.4512 |  |  |  |  |  |  | 0.02 |  |  |  |  |
| 257 | n-Hentriacontane; Untriacontane | 73.916 | C31H64 | 436.8399 | alkane |  |  |  |  |  |  |  | 0.02 |  |  |
| 258 | n-Pentatriacontane; n-Pentatriacotane | 73.948 | C35H72 | 492.9462 |  |  |  |  |  |  |  |  |  | 0.03 |  |
| 259 | Tetrapentacontane | 73.96 | C54H110 | 759.4512 |  |  |  |  |  |  |  |  |  |  | 0.04 |
| 260 | Tetrapentacontane | 74.046 | C54H110 | 759.4512 |  |  |  |  |  |  |  | 0.04 |  |  |  |
| 261 | 3-Methylheptadecane; Hexadecane, 2-ethyl | 74.057 | C18H38 | 254.4943 |  |  |  |  |  |  | 0.03 |  |  |  |  |
| 262 | trans-2-Dodecen-1-ol | 74.339 | C12H24O | 184.3184 |  |  | 0.11 |  |  |  |  |  |  |  |  |
| 263 | Nonadec-1-ene | 74.678 | C19H38 | 266.505 |  |  | 0.22 |  |  | 0.16 |  |  |  |  |  |
| 264 | Diallyldimethylsilane | 74.794 | C8H16Si | 140.2981 |  |  |  |  |  |  |  |  |  | 0.01 |  |
| 265 | Erucylamide; | 74.807 | C22H43NO | 337.5829 | amide |  |  |  |  |  |  |  | 0.02 |  |  |
| 266 | 1,2-Epoxyoctadecane; Hexadecyl oxirane | 75.083 | C18H36O | 268.4778 |  |  |  |  |  |  | 0.03 |  |  |  |  |
| 267 | n-Dotriacontane; Bicetyl | 75.116 | C32H66 | 450.8664 |  |  |  |  | 0.16 |  |  |  |  |  |  |
| 268 | n-Nonacosane | 75.129 | C29H60 | 408.7867 | alkane | 0.19 |  |  |  |  |  |  |  |  |  |
| 269 | n-Tetracontane | 75.157 | C40H82 | 563.0791 |  |  |  | 0.51 |  | 7 |  |  |  |  |  |
| 270 | n-Tetracontane | 75.214 | C40H82 | 563.0791 |  |  | 6.27 |  |  |  |  |  |  |  |  |
| 271 | Squalene | 75.767 | C30H50 | 410.718 | triter |  |  |  |  |  | 0.5 | 0.18 | 0.3 | 0.2 | 0.32 |
| 272 | 2-Hexyl-1-decanol; 2-Hexyldecanol | 76.075 | C16H34O | 242.4406 |  |  | 0.09 |  |  |  |  |  |  |  |  |
| 273 | Hexadecamethylheptasiloxane | 77.136 | C16H48O6Si7 | 533.1472 |  |  |  |  |  |  |  | 0.08 |  |  |  |
| 274 | Stearaldehyde; Octadecyl aldehyde | 77.135 | C18H36O | 268.4778 |  |  |  |  |  | 0.07 |  |  |  |  |  |
| 275 | Octadeamethyl-cyclononasiloxane | 77.576 | C18H54O9Si9 | 667.3855 |  |  |  |  |  |  |  |  | 3.87 | 4.64 | 3.43 |
| 276 | Arachidonic acid methyl ester;Methyl arachidonate | 77.595 | C21H34O2 | 318.4935 |  |  |  |  | 0.03 |  |  |  |  |  |  |
| 277 | Hexadecylene oxide; 1,2-Epoxyhexadecane | 77.598 | C16H32O | 240.4247 |  |  | 0.06 |  |  |  |  |  |  |  |  |
| 278 | Octadeamethyl-cyclononasiloxane | 77.61 | C18H54O9Si9 | 667.3855 |  | 0.05 |  | 6.19 |  | 0.46 | 4.66 | 6.21 |  |  |  |
| 279 | Linolenic acid; α-Linolenic acid; | 78.087 | C18H30O2 | 278.4296 |  |  |  |  | 0.03 |  |  |  |  |  |  |
| 280 | n-Nonacosane | 79.364 | C29H60 | 408.7867 | alkane |  |  |  |  |  |  |  |  |  | 0.29 |

Table S1. Continued

| N | Compound name | R.T | Formula | M.W/Da | Terpene of component | *W.T* | | | | | *SgCINS* | | | | |
| --- | --- | --- | --- | --- | --- | --- | --- | --- | --- | --- | --- | --- | --- | --- | --- |
|  |  |  |  |  |  | 0 h | 3 h | 6 h | 12 h | 24 h | 0 h | 3 h | 6 h | 12 h | 24 h |
| 281 | n-Dotriacontane; Bicetyl | 79.537 | C32H66 | 450.8664 |  |  |  |  |  | 0.08 |  |  |  |  |  |
| 282 | n-Tetracontane | 79.478 | C40H82 | 563.0791 |  |  |  |  |  |  | 0.51 | 0.39 | 0.19 | 0.21 |  |
| 283 | Tetrapentacontane | 79.559 | C54H110 | 759.4512 |  |  |  | 0.05 |  |  |  |  |  |  |  |
|  | Total percentage ( % ) of  component |  |  |  |  | 100 | 100 | 100 | 100 | 100 | 100 | 100 | 100 | 100 | 100 |
|  | Total percentage ( % ) of monoterpenes |  |  |  |  |  |  |  |  |  | 0.16 | 0.27 | 0.15 | 0.13 | 0.24 |
|  | Total percentage ( % ) of sesquiterpenes |  |  |  |  | 6.05 | 5.85 | 4.4 | 9.55 | 10.38 | 17.11 | 0.15 | 25.88 | 15.71 | 22.37 |
|  | Total percentage ( % ) of diterpenes |  |  |  |  | 16.1 | 21.32 | 15.15 | 27.25 | 7.44 | 3.6 | 3.91 | 6.33 | 3.69 | 5.14 |
|  | Total percentage ( % ) of triterpenes |  |  |  |  | 0.27 | 0.39 | 0.67 |  | 0.68 | 0.18 | 0.3 | 0.41 | 0.68 | 0.27 |
|  | Total percentage ( % ) of tetraterpene |  |  |  |  |  |  |  |  |  |  | 0.7 |  |  |  |
|  | Total percentage ( % ) of alkane |  |  |  |  | 0.19 |  |  |  |  |  |  | 0.09 |  | 0.01 |
|  | Total percentage ( % ) of ester |  |  |  |  | 0.48 | 0.81 | 0.52 | 0.7 |  | 1.15 | 0.41 | 0.84 | 0.49 | 0.94 |
