# Supplementary material for: Cloning and Characterization of 1,8-Cineole Synthase (SgCINS) Gene From the Leaves of Salvia guaranitica Plant
Source: Front Plant Sci. 2022 Apr 15;13:869432. doi: 10.3389/fpls.2022.869432 (PMC9051517; doi:10.3389/fpls.2022.869432)
Supplement: Supplementary file 2 [file Presentation_1.PPT]

## Slide 1
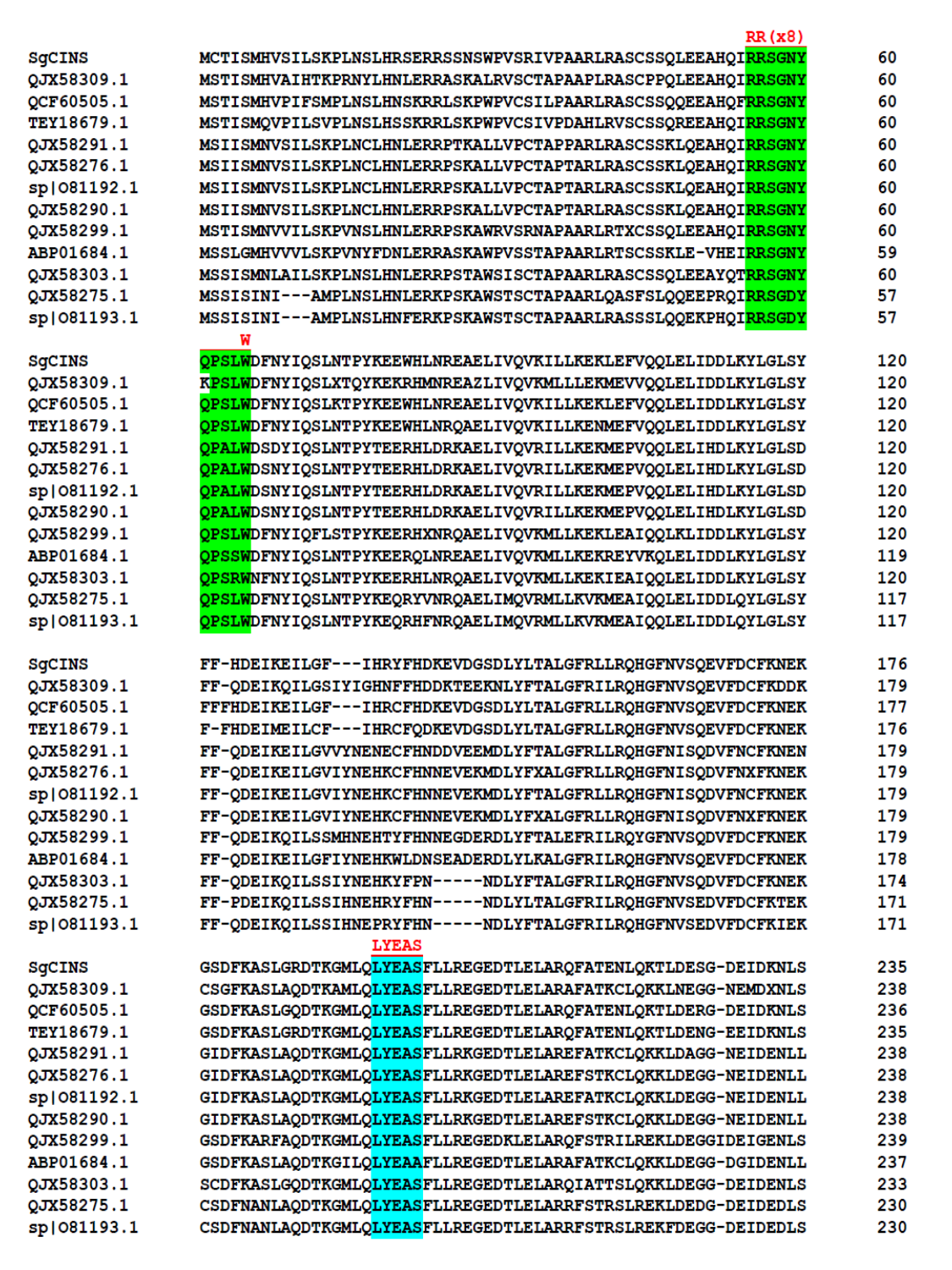

## Slide 2
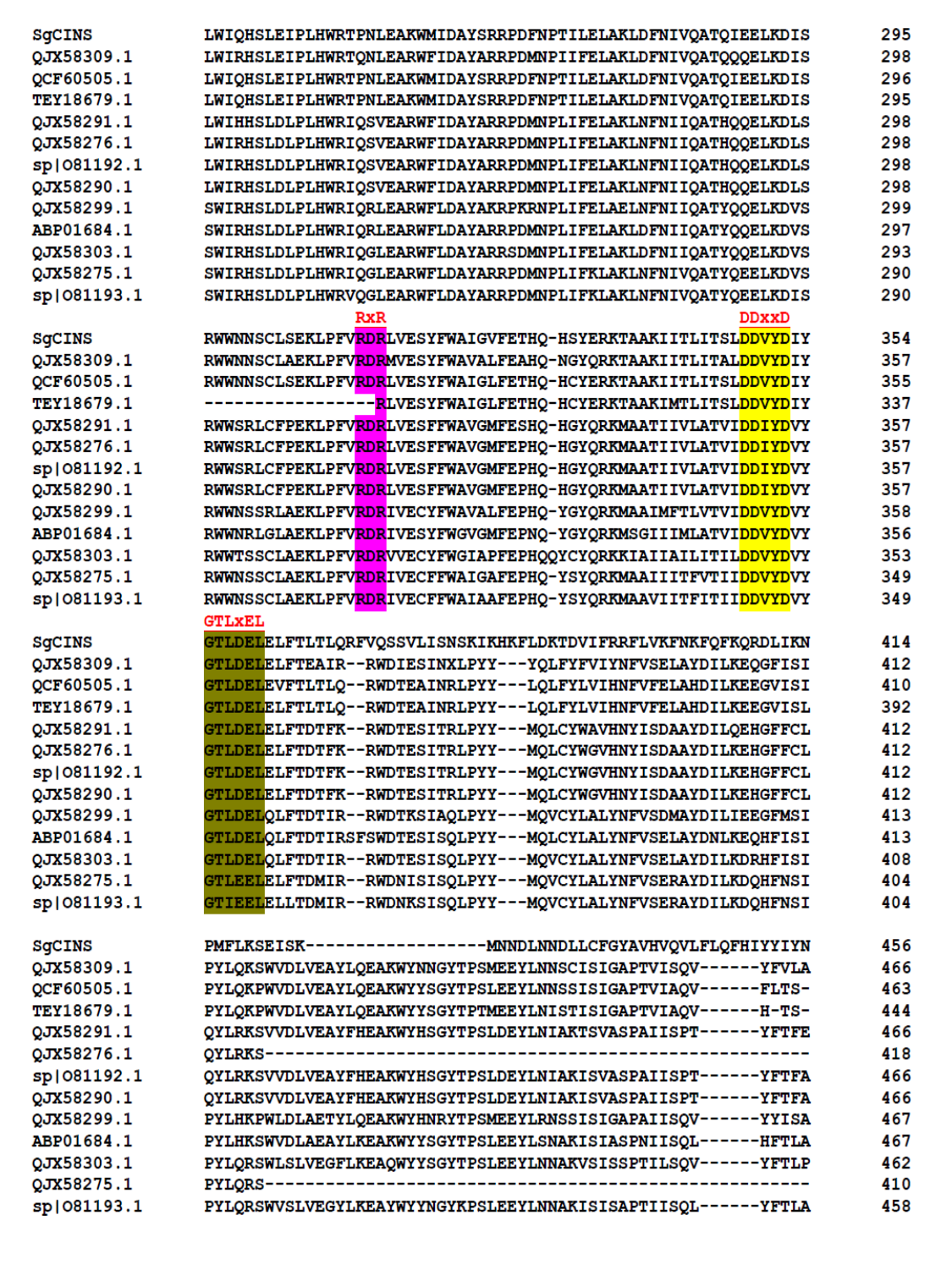

## Slide 3
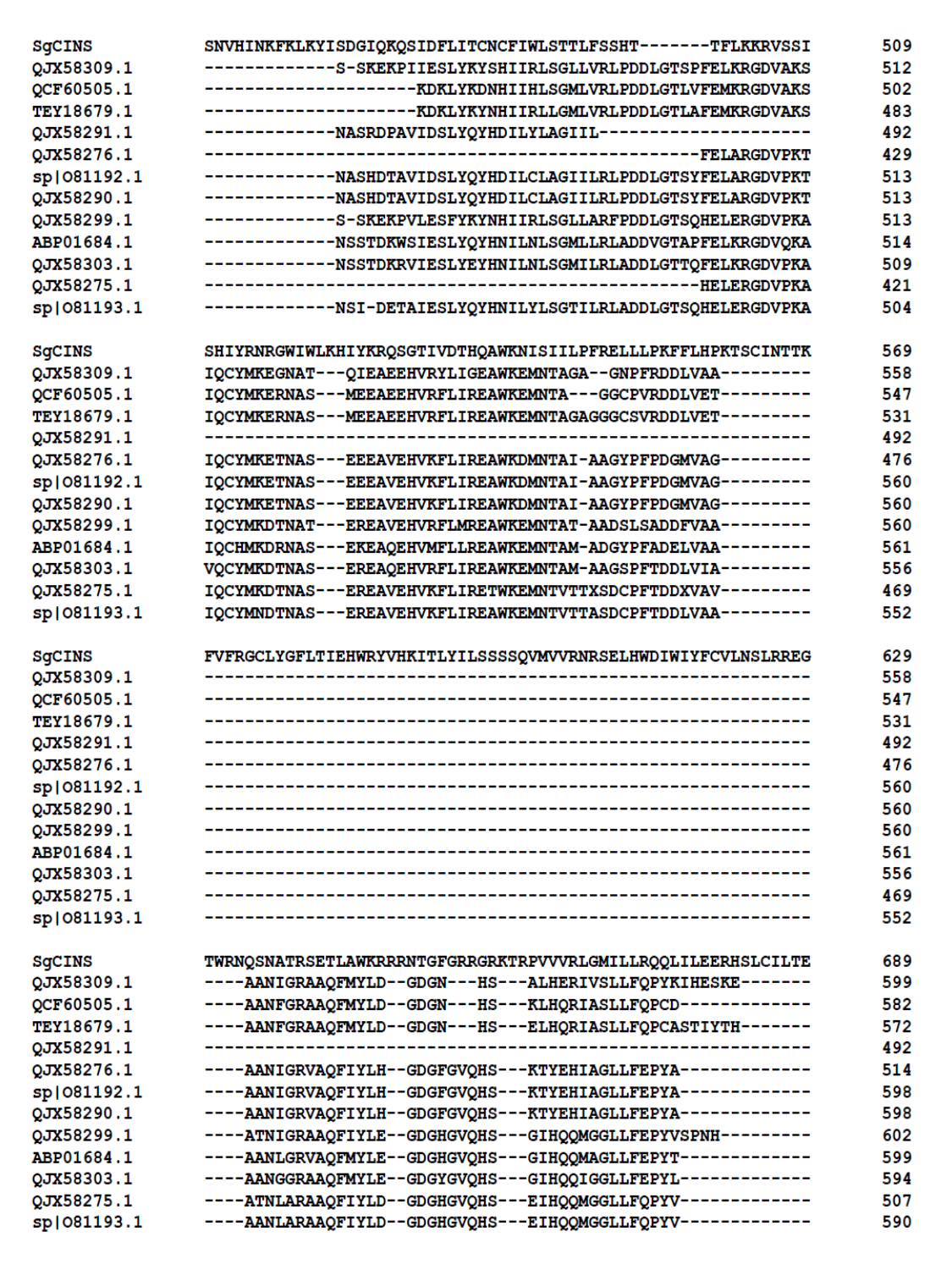

## Slide 4
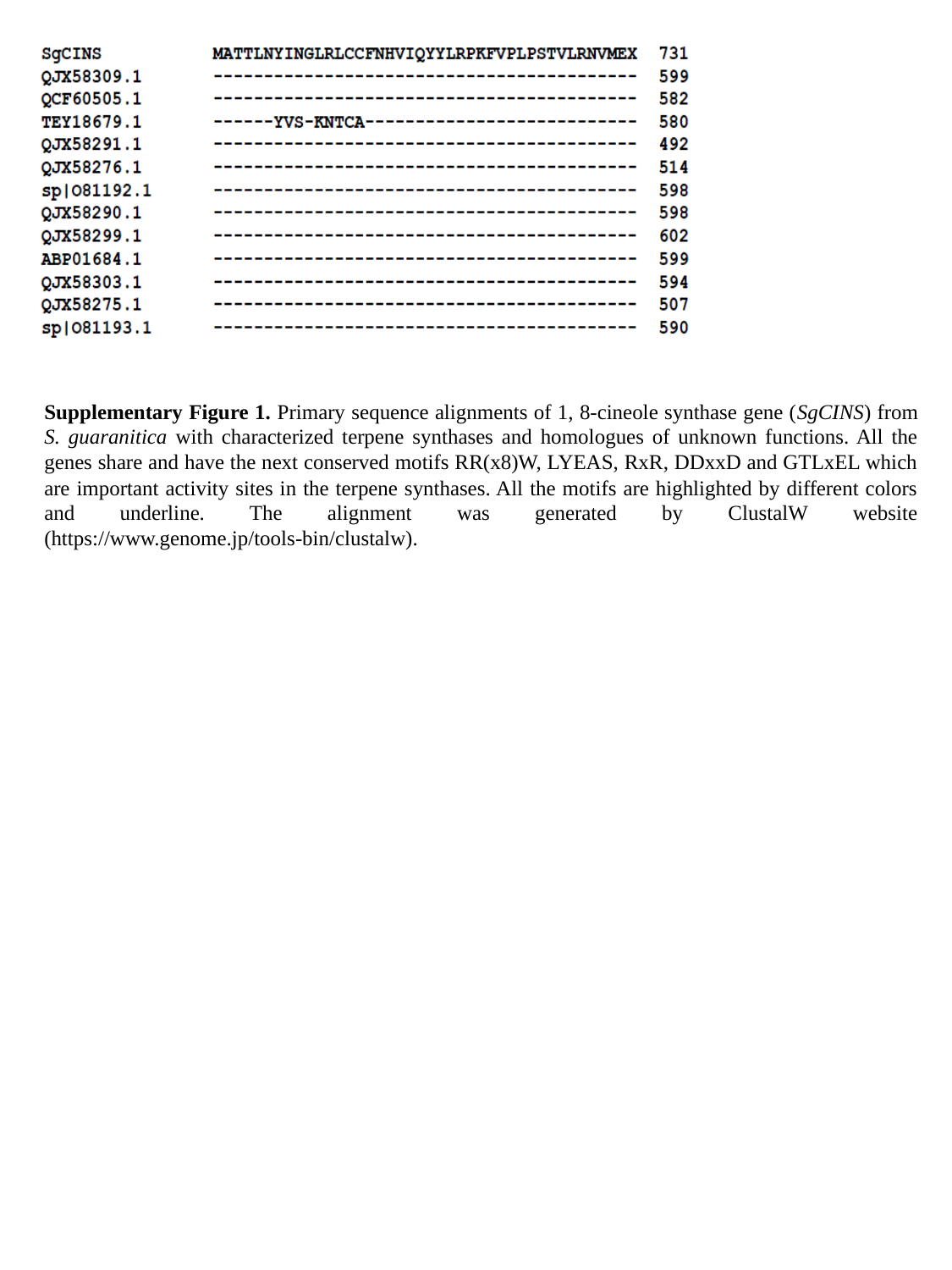

Supplementary Figure 1. Primary sequence alignments of 1, 8-cineole synthase gene (SgCINS) from S. guaranitica with characterized terpene synthases and homologues of unknown functions. All the genes share and have the next conserved motifs RR(x8)W, LYEAS, RxR, DDxxD and GTLxEL which are important activity sites in the terpene synthases. All the motifs are highlighted by different colors and underline. The alignment was generated by ClustalW website (https://www.genome.jp/tools-bin/clustalw).

## Slide 5
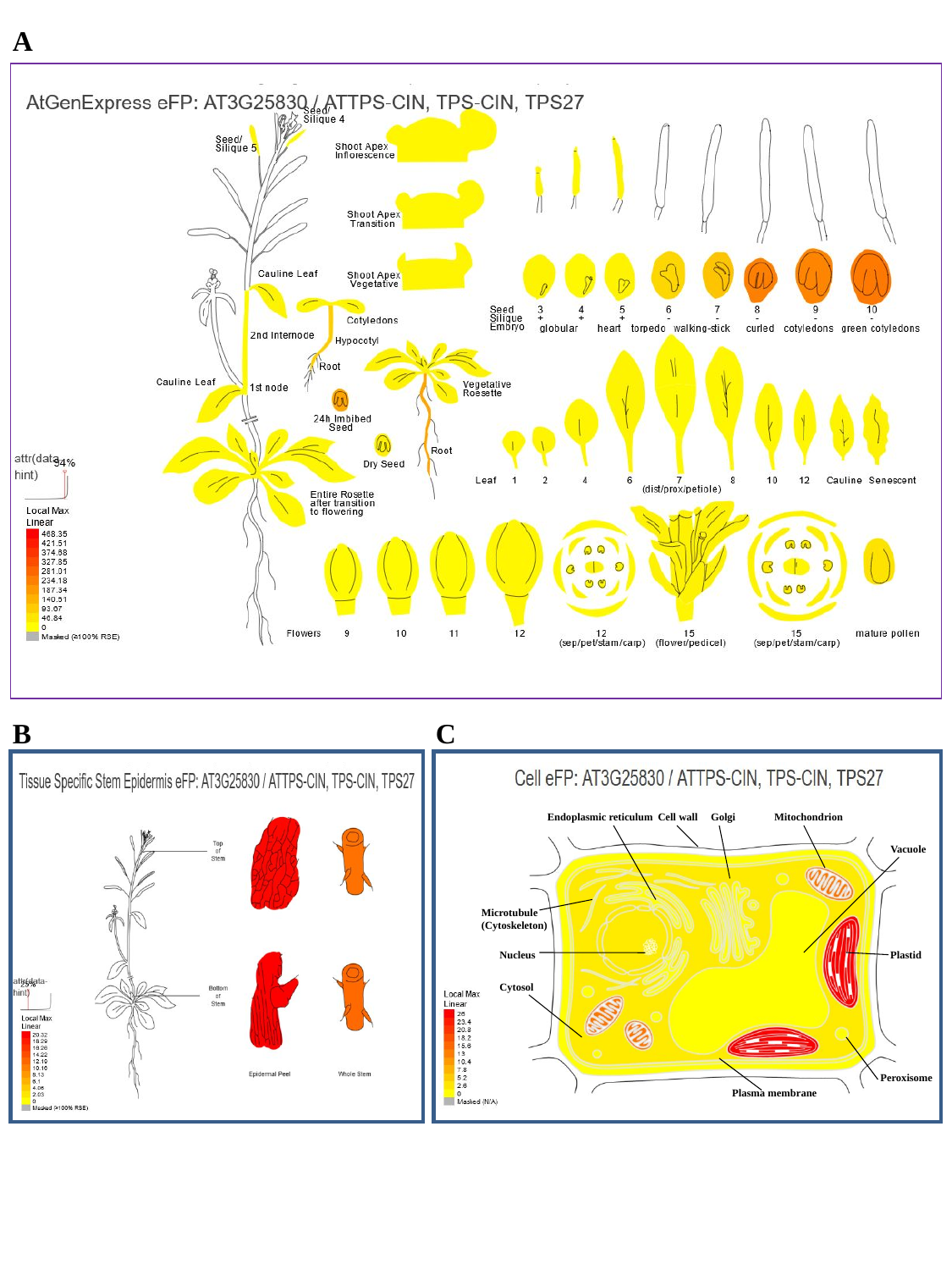

A
B
C
Endoplasmic reticulum
Cell wall
Golgi
Mitochondrion
Vacuole
Microtubule
(Cytoskeleton)
Nucleus
Plastid
Cytosol
Peroxisome
Plasma membrane

## Slide 6
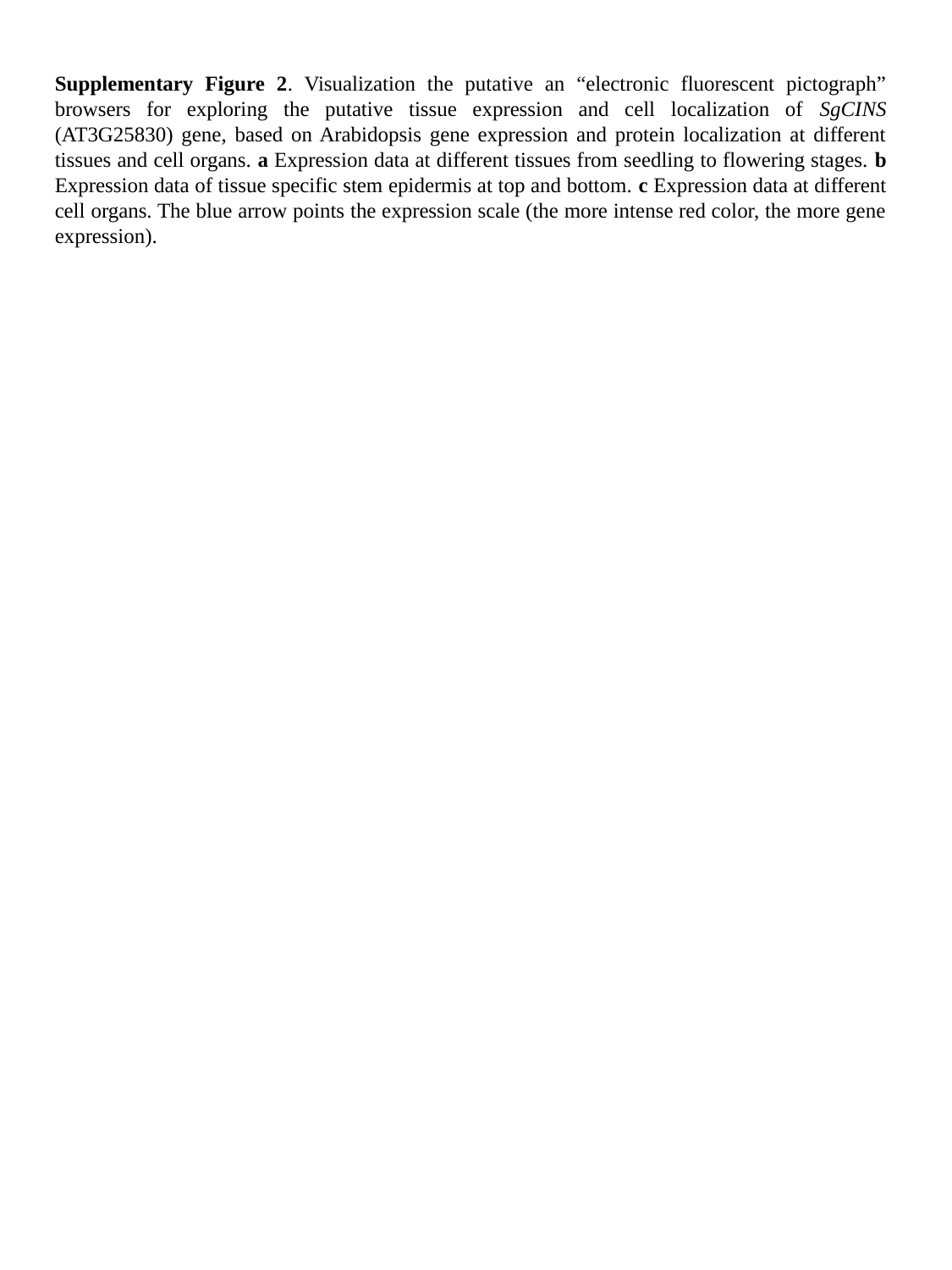

Supplementary Figure 2. Visualization the putative an “electronic fluorescent pictograph” browsers for exploring the putative tissue expression and cell localization of SgCINS (AT3G25830) gene, based on Arabidopsis gene expression and protein localization at different tissues and cell organs. a Expression data at different tissues from seedling to flowering stages. b Expression data of tissue specific stem epidermis at top and bottom. c Expression data at different cell organs. The blue arrow points the expression scale (the more intense red color, the more gene expression).

## Slide 7
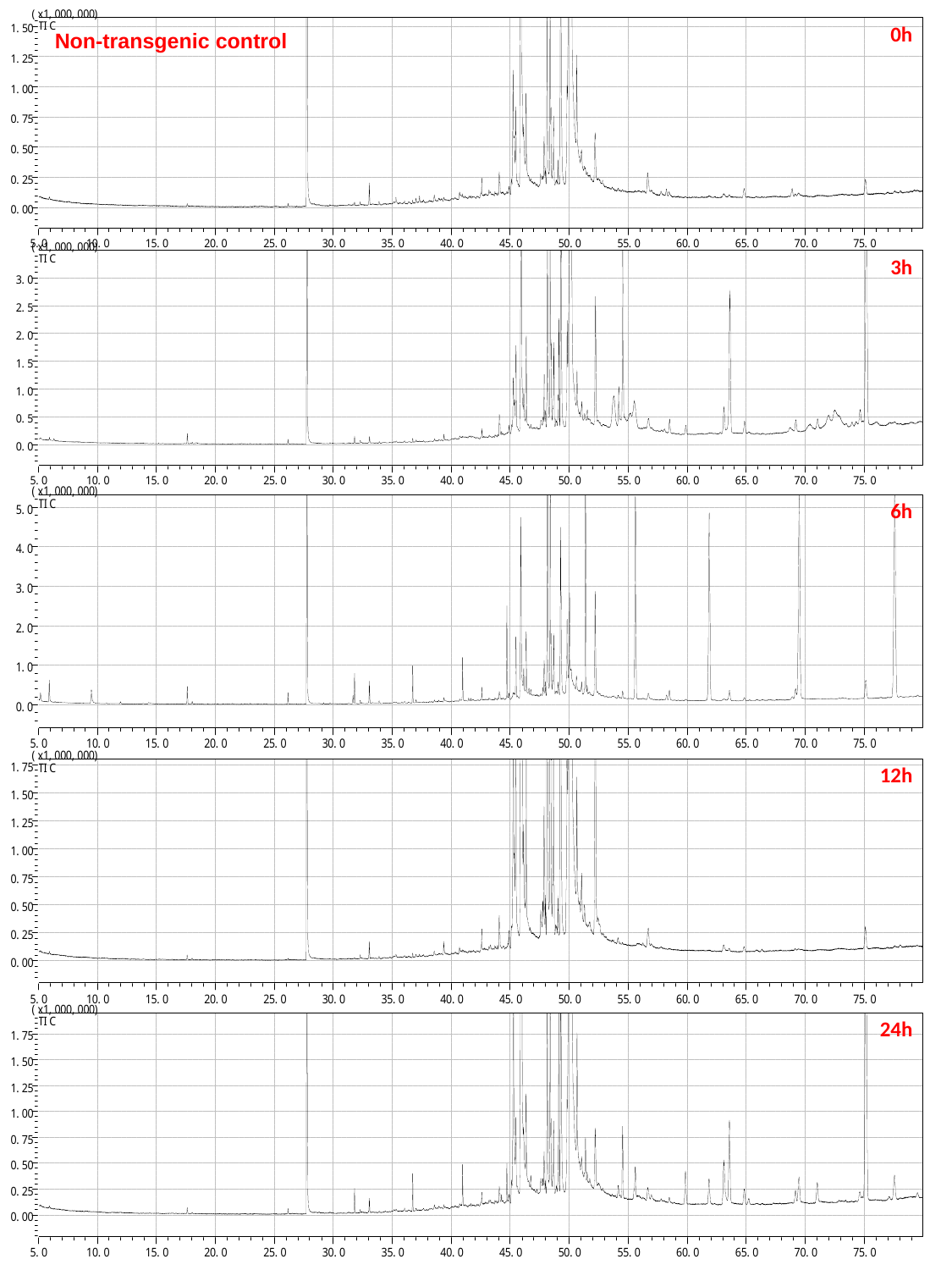

0h
Non-transgenic control
3h
6h
12h
24h

## Slide 8
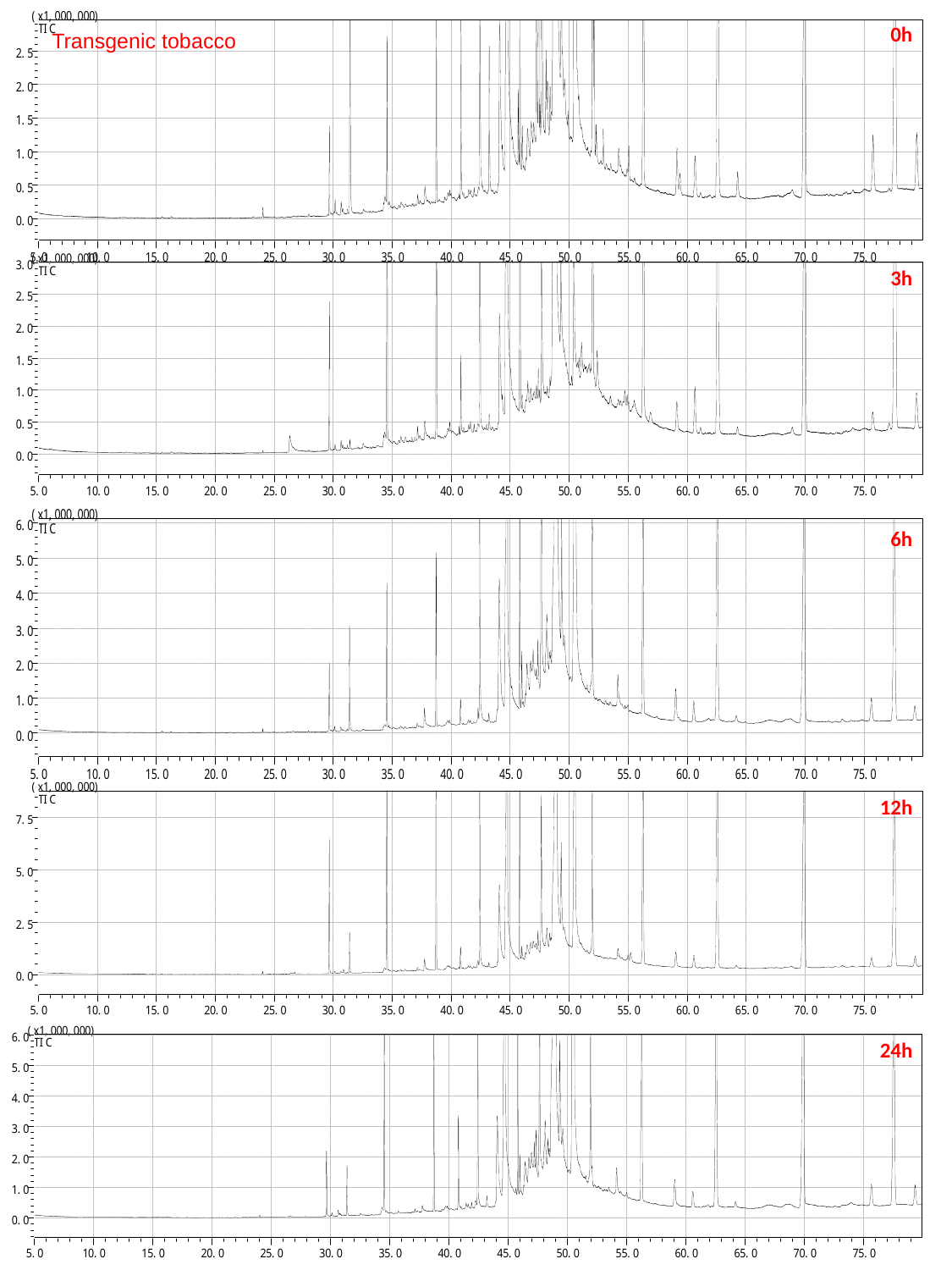

0h
Transgenic tobacco
3h
6h
12h
24h

## Slide 9
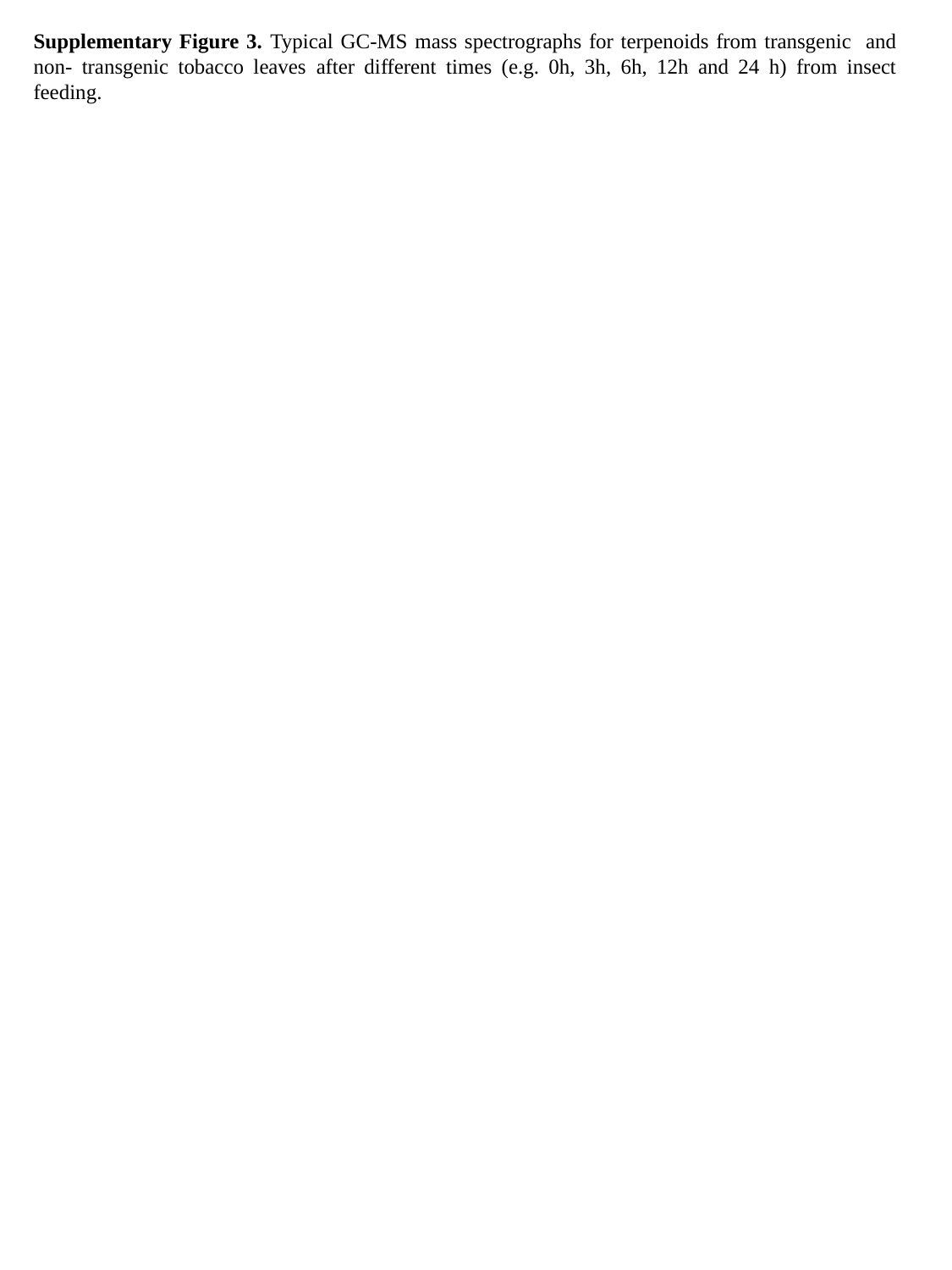

Supplementary Figure 3. Typical GC-MS mass spectrographs for terpenoids from transgenic and non- transgenic tobacco leaves after different times (e.g. 0h, 3h, 6h, 12h and 24 h) from insect feeding.
